# Dominant-acting *CSF1R* variants cause microglial depletion and altered astrocytic phenotype in zebrafish and adult-onset leukodystrophy

**DOI:** 10.1007/s00401-022-02440-5

**Published:** 2022-06-17

**Authors:** Woutje M. Berdowski, Herma C. van der Linde, Marjolein Breur, Nynke Oosterhof, Shanice Beerepoot, Leslie Sanderson, Lieve I. Wijnands, Patrick de Jong, Elisa Tsai-Meu-Chong, Walter de Valk, Moniek de Witte, Wilfred F. J. van IJcken, Jeroen Demmers, Marjo S. van der Knaap, Marianna Bugiani, Nicole I. Wolf, Tjakko J. van Ham

**Affiliations:** 1 Department of Clinical Genetics, Erasmus MC, University Medical Center Rotterdam, PO Box 2040, 3000 CA Rotterdam, The Netherlands; 2 Department of Child Neurology, Amsterdam Leukodystrophy Center, Emma Children’s Hospital, Amsterdam University Medical Centers, Vrije Universiteit, De Boelelaan 1117, 1081 HV Amsterdam, The Netherlands; 3 Amsterdam Neuroscience, Amsterdam University Medical Centers, Vrije Universiteit, De Boelelaan 1117, 1081 HV Amsterdam, The Netherlands; 4 Department of Pathology, Neuroscience Campus Amsterdam, VU University Medical Center, Amsterdam, The Netherlands; 5 European Research Institute for the Biology of Ageing (ERIBA), University of Groningen, University Medical Center Groningen, Groningen, The Netherlands; 6 Hematology Department, University Medical Center, Utrecht, The Netherlands; 7 Center for Biomics, Erasmus MC University Medical Center, Rotterdam, The Netherlands; 8 Proteomics Center, Erasmus University Medical Center, Wytemaweg 80, 3015 CN Rotterdam, The Netherlands

**Keywords:** Microglia, CSF1R, Leukodystrophy, ALSP, Zebrafish models, Astrocytes

## Abstract

Tissue-resident macrophages of the brain, including microglia, are implicated in the pathogenesis of various CNS disorders and are possible therapeutic targets by their chemical depletion or replenishment by hematopoietic stem cell therapy. Nevertheless, a comprehensive understanding of microglial function and the consequences of microglial depletion in the human brain is lacking. In human disease, heterozygous variants in *CSF1R*, encoding the Colony-stimulating factor 1 receptor, can lead to adult-onset leukoencephalopathy with axonal spheroids and pigmented glia (ALSP) possibly caused by microglial depletion. Here, we investigate the effects of ALSP-causing *CSF1R* variants on microglia and explore the consequences of microglial depletion in the brain. In intermediate- and late-stage ALSP post-mortem brain, we establish that there is an overall loss of homeostatic microglia and that this is predominantly seen in the white matter. By introducing ALSP-causing missense variants into the zebrafish genomic *csf1ra* locus, we show that these variants act dominant negatively on the number of microglia in vertebrate brain development. Transcriptomics and proteomics on relatively spared ALSP brain tissue validated a downregulation of microglia-associated genes and revealed elevated astrocytic proteins, possibly suggesting involvement of astrocytes in early pathogenesis. Indeed, neuropathological analysis and in vivo imaging of *csf1r* zebrafish models showed an astrocytic phenotype associated with enhanced, possibly compensatory, endocytosis. Together, our findings indicate that microglial depletion in zebrafish and human disease, likely as a consequence of dominant-acting pathogenic *CSF1R* variants, correlates with altered astrocytes. These findings underscore the unique opportunity *CSF1R* variants provide to gain insight into the roles of microglia in the human brain, and the need to further investigate how microglia, astrocytes, and their interactions contribute to white matter homeostasis.

**Supplementary Information:**

The online version contains supplementary material available at 10.1007/s00401-022-02440-5.

## Introduction

Tissue-resident macrophages, including microglia, play a significant role in shaping brain development and connectivity as well as maintaining normal brain function [8, 24, 49, 67, 80, 86, 100, 111, 119]. Microglia in particular have been implicated in the pathogenesis of neurodegenerative diseases, including Alzheimer’s disease (AD) and multiple sclerosis (MS) [70, 89, 94, 120]. Strikingly, genetic variants in several microglia-specific genes cause ‘microgliopathies’ [94], a subgroup of leukodystrophies, which are defined as central nervous system (CNS) disorders primarily involving brain white matter. In both neurodegenerative diseases and microgliopathies, microglia increasingly gain interest as potential therapeutic targets, as they can be repopulated using hematopoietic stem cell transplantation (HSCT) or could be pharmacologically depleted by Colony-stimulating factor 1 receptor (CSF1R) inhibitors [17, 106, 107, 123]. It is, therefore, critical to establish a better understanding of microglial function and the consequences of microglial depletion for the human brain.

CSF1R is a protein primarily located on mononuclear phagocytes and considered a key regulator of macrophage and microglial biology [50, 110]. Bi-allelic variants in *CSF1R* can cause a severe, but variable, developmental disorder termed BANDDOS (brain abnormalities, neurodegeneration, and dysosteosclerosis) [40, 55, 74, 81, 114]. The few patients described presented with dysosteosclerosis, severe white matter lesions and other congenital brain abnormalities, leading to early death [40, 74, 81]. Analysis of post-mortem brain tissue of a patient with a homozygous variant causing disruption of a splice acceptor site showed brain calcifications, axonal spheroids and an almost complete absence of microglia [81]. Similarly, loss of CSF1R in rats, mice and zebrafish causes largely halted macrophage/microglia development, resulting in skeletal and CNS abnormalities [29, 34, 62, 91]. Heterozygous missense variants located in the tyrosine kinase domains (TKDs) of CSF1R have been found to cause the more prevalent adult-onset leukodystrophy, termed ALSP (adult-onset leukoencephalopathy with axonal spheroids and pigmented glia) [95]. Hallmarks of ALSP are motor and cognitive functional decline, often leading to death within several years after disease onset, and neuropathologically progressive white matter lesions, axonal spheroids, pigmented glia and cerebral calcifications [1, 11, 121]. Recent evidence showed that heterozygous *CSF1R* variants may also be associated with lower density of microglia in ALSP patients [83, 85, 113]. HSCT, which may act by supplying bone-marrow-derived cells that repopulate the microglial niche, was suggested for treatment of ALSP. In mice, HSCT is indeed capable of repopulating the microglial niche, but only after depletion of endogenous microglia [9]. More recently, it was shown that HSCT can halt disease progression in ALSP patients and even reduce pathology visible on MRI [39, 73]. It is possible that the lack of microglia in ALSP facilitates their re-introduction by HSCT. Nevertheless, it remains not fully established whether microglial depletion is the initial pathological event in ALSP, which is important in understanding the mechanisms underlying the highly beneficial effects of HSCT.

Intriguingly, parents of patients with bi-allelic variants, even those with complete loss of function, have not been noted to develop ALSP or neurological dysfunction [40, 55, 74, 81, 114]. While parents of patients with bi-allelic variants mostly have frameshift variants leading to nonsense-mediated mRNA decay (NMD), most ALSP-causing CSF1R variants are missense and located in the TKDs. This is consistent with a model where missense variants act dominantly and, at the biochemical level, cause dysfunctional heterodimers of wild-type and mutant CSF1R, which fail to show tyrosine kinase activity and lead to a ~ 75% suppression of CSF1R activity [50,^92^]. This is also consistent with findings in haploinsufficient *Csf1r*-mutant mice where microglia were not reduced [7, 19]. However, support for a dominant effect of ALSP-causing missense variants in vivo is lacking. Gaining insight into how these variants affect microglia, and their subsequent effect on brain health, is crucial to establish and refine treatment strategies for ALSP while also aiding in the development of microglia-focused therapies for other CNS diseases.

Here, we investigated the effects of ALSP-causing *CSF1R* variants on microglia and pathology correlating with microglial depletion, which could precede white matter degeneration. We integrated neuropathological and multi-omic analysis on post-mortem brain tissue of ALSP patients with in vivo applications in genetic zebrafish models. Zebrafish have been used extensively to study developmental genetics and function of microglia, as they show highly conserved basic properties including CSF1R function [45, 46, 62, 67, 81, 82, 88, 104, 105]. We provide evidence for microglial depletion both in ALSP post-mortem tissue and in vivo in zebrafish with pathogenic *CSF1R* variants. Furthermore, we identified an unexpected astrocytic phenotype correlating with microglial depletion both in zebrafish models and in ALSP patients. Altogether, our data show that microglial depletion caused by dominant *CSF1R* variants is a key hallmark and initial pathological event in ALSP. This further supports why HSCT in ALSP patients would be beneficial, possibly by repopulation of the microglial niche.

## Materials and methods

### Human brain tissue

Post-mortem human brain tissue (paraffin-embedded and fresh–frozen) of the cingulate gyrus, frontal gyrus and occipital gyrus of two late-stage ALSP donors and two non-demented age- and sex-matched controls was provided by the Netherlands Brain Bank (NBB, Amsterdam, The Netherlands, https://www.brainbank.nl/). Four patients diagnosed with pigmentary orthochromatic leukodystrophy (POLD), later found to be the same disease currently known as ALSP, were generously provided by JM Powers of the Rochester University, Rochester, New York [69]. In addition, post-mortem brain tissue of 2 intermediate-stage ALSP patients was provided by the Amsterdam Leukodystrophy Center. Informed consent was obtained from donors for brain autopsy and the use of tissue and clinical information for research purposes. Demographic and clinical information of late-stage ALSP patients 1 and 2 has been described previously and here summarized in Supplementary Table 1, Online Resources [83]. Clinical information on four patients diagnosed with POLD are summarized in Supplementary Table 1, Online Resources. Demographic and clinical information of intermediate-stage ALSP patients is provided below.

### Intermediate-stage patients

**Fig. 1:**
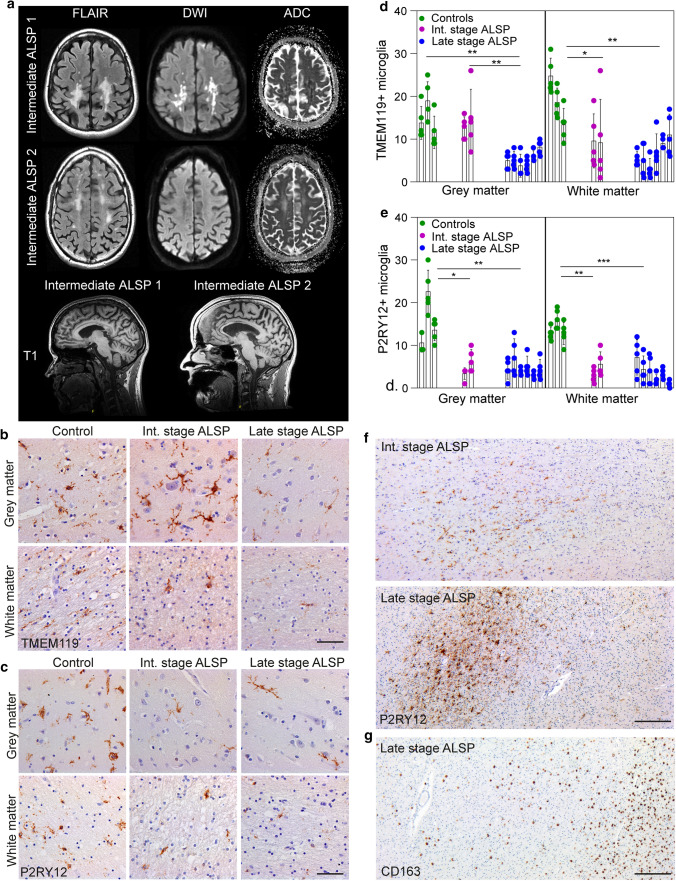
ALSP patients show an overall loss of (homeostatic) microglia and altered microglial distribution. **a** Brain MRI abnormalities in intermediate-stage ALSP patient 1 and intermediate-stage ALSP patient 2. Axial FLAIR images show hyperintense white matter abnormalities, most pronounced in the bilateral parietal and occipital lobes of intermediate-stage ALSP patient 1 and in the bilateral parietal and frontal lobes of intermediate-stage ALSP patient. Hyperintense signals on diffusion-weighted imaging (DWI) and corresponding low signal on apparent diffusion coefficient (ADC)-maps indicate restricted diffusion in the majority of the lesions in patient 1, but not in patient 2. Sagittal T1-weighted images show corpus callosum involvement and mild generalized brain atrophy in both patients. **b** Representative TMEM119 IHC images of the frontal gyrus of controls (*n* = 3), intermediate-stage ALSP patients (*n* = 2) and late-stage ALSP patients (*n* = 6) in grey matter (top row) and white matter (bottom row). **c** Representative P2RY12 IHC images of the frontal gyrus of controls (*n* = 3), intermediate-stage ALSP patients (*n* = 2) and late-stage ALSP patients (*n* = 6) in grey (top row) and white matter (bottom row). **d, e** Quantification of TMEM119 + (**d**) and P2RY12 + (**e**) microglia in grey (left) and white matter (right) of frontal gyrus. Data points represent microglia in one randomly taken image, 5 images/individual. **f** Representative P2RY12 IHC images of the frontal gyrus showing clustered distribution of P2RY12 + microglia in the white matter. **g** Representative CD163 IHC images of the frontal gyrus of late-stage ALSP (*n* = 6) patients showing clustered distribution of CD163 + cells in the white matter. Int.: intermediate. A nested one-way ANOVA test was preformed to test for significance (*p* < 0.05). Error bars represent SD. *p < 0.05, **p < 0.01, ***p < 0.001. Scale bars equal 50 μm (**b**, **c**) and 500 μm (**f**, **g**)

Intermediate-stage ALSP patient 1 (female) was diagnosed at age 31 years with progressive upper and lower limb weakness, impaired balance and fine motor skills, facial asymmetry and pain complaints since the age of 30 years. MRI showed T2 hyperintense white matter abnormalities, most pronounced in the splenium of the corpus callosum and in the bilateral parietal lobes extending to the occipital lobes and generalized cerebral atrophy (Fig. 1a). DWI showed diffusion restriction in the majority of the lesions (Fig. 1a). A heterozygous pathogenic variant *c.2330G* > *A* p.(Arg777Gln) in *CSF1R* confirmed the diagnosis of ALSP. Since the patient was still able to walk and had relatively intact cognitive function, disease stage in this patient was classified as intermediate-stage ALSP. Because of rapid decline, palliative care was initiated and she passed away at the age of 32 years.

Intermediate-stage ALSP patient 2 (male) was diagnosed at age 44 years by family screening due to dominant heterozygous pathogenic variant c.2329C > T, p.(Arg777Trp). His clinical signs developed at age 47.9 years and consisted of impaired balance, rigidity, tremor, memory deficits and slowness of thinking. One year later, his MRI revealed typical T2 hyperintense white matter abnormalities in the bilateral parietal and frontal lobes with corpus callosum involvement and mild generalized cerebral atrophy, but no areas of restricted diffusion in the white matter were present (Fig. 1a). His cognitive and motor functioning on exam were moderately impaired, indicating an intermediate disease stage. He received palliative care and passed away at the age of 49 years.

### Animal models

Zebrafish were maintained under standard conditions [4]. Adult animals were fed brine shrimp twice a day and kept in groups on a 14-h light and 10-h dark cycle. Zebrafish embryos and larvae (until 5 days post-fertilization (dpf)) were kept at 28 °C on a 14–10‐h light–dark cycle in 1 M HEPES buffered (pH 7.2) E3 medium (34.8 g NaCl, 1.6 g KCl, 5.8 g CaCl_2_·2H_2_O, 9.78 g MgCl_2_·6H_2_O). Before 24 h post-fertilization (hpf), the medium was changed to 0.003% 1‐phenyl 2‐thiourea (PTU) in E3 to prevent pigmentation.

The following mutant zebrafish lines were used: wild-type AB, *csf1ra*^j4e1/j4e1^ with a Val614Met substitution in the first TKD [87], *csf1ra*^*re25/re25*^ carrying the Ala784Val substitution in exon 17 in the second TKD (*csf1ra*^*A784V/A784V*^), *csf1ra*^*re26/re26*^ (*csf1ra*^*ex17Δ5/ex17Δ5*^) with a 5 bp deletion and premature stop in exon 17 in the second TKD, *csf1rb*^re01/re01^ with a 4 bp deletion and premature stop codon in exon 3 [83]. All *csf1ra* mutants were crossed with *csf1rb*^re01/re01^ to generate *csf1ra* mutants in a *csf1rb*-deficient background, to avoid compensation of the *csf1rb* gene. The *csf1ra*^j4e1/j4e1^ and *csf1ra*^*re25/re25*^ mutants in a *csf1rb-*deficient background were then crossed with Tg(*mpeg1*:*eGFP*) zebrafish expressing GFP under the control of the *mpeg1* promotor [33]. In addition, *csf1ra*^j4e1/j4e1^ fish in a *csf1rb*-deficient background were crossed with the following transgenic lines: tgBAC(*slc1a2b*:*Citrine*) (re10Tg) fish expressing cytosolic Citrine under the control of the *slc1a2b* promotor [61], Tg(*her4*:*EGFP*) [124], Tg(*her4.1*:*mCherryT2ACreER*^*T2*^) [60], Tg(*sox10*:*RFP*) [56], Tg(*ubi*:*secA5*-*mVenus*) (plasmid was provided by Dr. Marco Morsch, Macquarie University, Sydney [61,75]), Tg(*mbp*:*EGFP*-*CAAX*) [27] and Tg(*elavl3*:GCaMP5G) [3]. Animal keeping was approved by the Animal Experimentation Committee at Erasmus MC, Rotterdam.

## Human donor examination

### Immunohistochemistry (IHC) human brain tissue

For neuropathological examination of axonal spheroids, myelin, microglia and astrocyte, paraffin-embedded post-mortem brain tissue sections were cut (4 µm) using a microtome. In short, paraffin sections were deparaffinized and rehydrated to distilled water. Antigens were retrieved by heating sections in sodium citrate buffer (10 mM; pH 6.0). For horseradish peroxidase (HRP) staining, endogenous peroxidase was quenched in 0.2% H_2_O_2_/0.125 sodiumazide in 1 × phosphate-buffered saline (PBS) for a maximum of 30 min at room temperature (RT). Sections were washed in blocking buffer (PBS, 0.5% protifar (Nutricia), 0.15% glycine (Sigma-Aldrich, St. Louis, USA), 0.4% Tween20). Then, sections were incubated overnight at 4 °C with blocking buffer containing primary anti-bodies: rabbit anti-TMEM119 (Atlas, HPA051870; 1:500), rabbit anti-P2RY12 (Anaspec. AS55042a; 1:500), rabbit anti-ALDH1L1 (Sigma, HPA031332, 1:500), rabbit anti-GFAP (Sigma, G9269; 1:100), mouse anti-S100β (Abcam, AB218513; 1:100), rabbit anti-MBP (Sigma, M3821; 1:100), rabbit anti-GSTM1 (Proteintech, 12412-1-AP; 1:100) and rabbit anti-LAMP1 (Abcam, AB218513; 1:200). After washing with blocking buffer, sections were incubated with secondary anti-bodies (1:200): DyLight alexa 488 anti-rabbit (ThermoFisher, AB_2313584); DyLight alexa 488 anti-mouse (ThermoFisher, AB_2340846); DyLight alexa 647 anti-rabbit (ThermoFisher, AB_2492288), DyLight alexa 647 anti-mouse (ThermoFisher, AB_2340862) for 1 h at RT. Autofluorescence and background staining was blocked by incubating sections in Sudan Black solution for 3 min at RT. Sections were embedded in ProLong™ Gold Antifade Mountant with DAPI (Invitrogen, P36931) to visualize nuclei. For HRP staining, sections were incubated with goat anti-mouse/rabbit-HRP (ImmunoLogic, DPVO55HRP) for 1 h at RT, and subsequently developed with 3,3'-diaminobenzidine (DAB; 0.05 mg/ml, Sigma-Aldrich). After washing in distilled water, slides were counterstained with hematoxylin and eosin (HE) to visualize nuclei, dehydrated and embedded in Entellan (Merck).

### RNA sequencing

Total RNA was isolated from fresh–frozen occipital gyrus (mixed white matter and grey matter) of late-ALSP patient 1 and 2 (in triplicate) and 2 age- and sex-matched controls (in triplicate). Briefly, total RNA was isolated using TRIzol™ Reagent (ThermoFisher Scientific), 200 ng of total RNA was purified using poly-T oligo-attached magnetic beads to end up with poly-A containing mRNA. RIN values ranged from 5.5 to 6.5 for control and ALSP patient 1 RNA, and the RIN value was 7.9 for ALSP patient 2 RNA. The poly-A-tailed mRNA was fragmented and cDNA was synthesized using SuperScript II and random primers in the presence of Actinomycin D. cDNA fragments were end repaired, purified with AMPure XP beads, and A-tailed using Klenow exo-enzyme in the presence of dATP. Paired end adapters with dual index (Illumina) were ligated to the A-tailed cDNA fragments and purified using AMPure XP beads. The resulting adaptor-modified cDNA fragments were enriched by PCR using Phusion polymerase as followed: 30 s at 98 °C, 15 cycles of (10 s at 98 °C, 30 s at 60 °C, 30 s at 72 °C), 5 min at 72 °C. PCR products were purified using AMPure XP beads and eluted in 30 ml of resuspension buffer. One microliter was loaded on an Agilent Technologies 2100 Bioanalyzer using a DNA 1000 assay to determine the library concentration and for quality check.

Bridge amplification, sequencing by synthesis and data analysis Cluster generation was performed according to the Illumina TruSeq SR Rapid Cluster kit v2 (cBot) Reagents Preparation Guide (www.illumina.com). Briefly, 12 RNA-seq libraries were pooled together to get a stock of 10 nM. One microliter of the 10 nM stock was denaturated with NaOH, diluted to 6 pM and hybridized onto the flowcell. The hybridized products were sequentially amplified, linearized, and end-blocked according to the Illumina Single Read Multiplex Sequencing user guide. After hybridization of the sequencing primer, sequencing-by-synthesis was performed using the HiSeq 2500 with a single read 50-cycle protocol followed by dual index sequencing. Reads were aligned against the GRCh38 genome using HiSat2 (version 2.0.4) [53]. Counts were generated for each gene from the Ensembl (version 85) transcriptome analysis of GRCh38, using htseq-count (version 0.6.1) [5].

Differential gene expression analysis was performed using the Bioconductor package edgeR [98] and biomaRt [31, 32]. Differentially expressed genes were selected based on the following thresholds: log fold change (logFC) <1.5, FDR false-discovery rate (FDR) < 0.05 (Suppl. Table 3, Online Resources). Pathway analysis was performed using the Bioconductor package topGO (Suppl. Table 4, Online Resources). For further analysis, the DEG were compared to published gene sets to find overlapping genes, including human microglia [38], extracellular matrix-related genes [77], mouse and human oligodendrocyte-related genes [64, 66], MS microglia [122] and AD microglia [108] (Suppl. Table 6, Online Resources).

### Mass spectrometry

Protein lysates were obtained from fresh–frozen occipital gyrus (mixed white matter and grey matter) from late-ALSP patient 1 and 2 (1 in triplicate, 1 in duplicate) and 2 age- and sex-matched controls (1 in triplicate, 1 in duplicate). The brain tissue was cut and lysed in 1 ml 50 mM Tris/HCl pH 8.2, 0.5% sodium deoxycholate (SDC) and MS-SAFE™ protease and phosphatase inhibitor using a Bioruptor ultasonicator (Diagenode). Protein concentrations were measured using the BCA assay (Thermo Scientific). Lysates were reduced with 5 mM DTT and cysteine residues were alkylated with 10 mM iodoacetamide. Protein was extracted by acetone precipitation at − 20 °C overnight. Samples were centrifuged at 8000 *g* for 10 min at 4 °C. The acetone was removed and the pellet allowed to dry. The protein pellet (~ 4 mg protein) was dissolved in 1 ml 50 mM Tris/HCl pH 8.2, 0.5% SDC and proteins were digested with LysC (1:200 enzyme:protein ratio) for 4 h at 37 °C. Next, trypsin was added (1:100 enzyme:protein ratio) and the digestion proceeded overnight at 30 °C. Digests were acidified with 50 μl 10% formic acid (FA) and centrifuged at 8000 g for 10 min at 4 °C to remove the precipitated SDC. The supernatant was transferred to a new centrifuge tube. The digests were purified with C18 solid phase extraction (Sep-Pak, Waters), lyophilized and stored at − 20 °C.

Isobaric labeling of the enriched peptides was performed using the 10-plex tandem mass tag (TMT) reagents (Thermo Fisher Scientific) with some modifications to the method of Böhm et al. [13]. Peptides were loaded onto 20 mg C18 cartridges prepared in-house. The C18 cartridges were washed once with 1 ml 0.1% TFA and two times with 1 ml of 50 mM KH2PO4 (pH 4.5). TMT reagents (0.8 mg) were dissolved in 10 μl of dry ACN and diluted with 200 μl 50 mM KH2PO4. This TMT solution was immediately loaded onto the column and labeling on column proceeded for 1 h at RT. Each of the 9 samples was labeled with a different TMT tag. After labeling columns were washed twice with 1 ml 2% ACN/0.2% FA and the labeled peptides eluted with 1 ml 50% ACN. TMT-labeled samples were pooled and lyophilized.

TMT-labeled peptides were subjected to offline orthogonal high-pH reverse phase fractionation. TMT-labeled peptides were solubilized in 0.1% TFA and loaded onto a 20 mg PLRP-S cartridge made in-house. Cartridges were washed once with 1 ml 0.1% TFA and three times with 1 ml milliQ water. Peptides were eluted step-wise from column with 5%, 10%, 15% and 50% ACN/10 mM ammonium formate (pH 10). The 4 fractions were dried by vacuum centrifugation and each fraction was reconstituted with 2% ACN/0.2% FA for nLC-MS/MS analysis.

Mass spectra were acquired on an Orbitrap Lumos (Thermo) coupled to an EASY-nLC 1200 system (Thermo). Peptides were separated on an in-house packed 75 μm inner diameter column containing 50 cm Waters CSH130 resin (3.5 μm, 130 Å, Waters) with a gradient consisting of 2–20% (ACN, 0.1% FA) over 200 min at 300 nl/min. The column was kept at 50 °C in a NanoLC oven—MPI design (MS Wil GmbH). For all experiments, the instrument was operated in the data-dependent acquisition (DDA) mode. MS1 spectra were collected at a resolution of 120,000, with an automated gain control (AGC) target of 2E5 and a max injection time of 50 ms. The most intense ions were selected for MS/MS, top speed method 3-s cycle time. Precursors were filtered according to charge state (2–7), and monoisotopic peak assignment. Previously interrogated precursors were dynamically excluded for 70 s. Peptide precursors were isolated with a quadrupole mass filter set to a width of 1.2 Th. When applying the MS3 method, ion trap MS2 spectra were collected at an AGC of 5E4, max injection time of 50 ms and CID collision energy of 35%. For Orbitrap MS3 spectra, the operation resolution was 60,000, with an AGC setting of 1E5 and a max injection time of 120 ms. The HCD collision energy was set to 65% to ensure maximal TMT reporter ion yield. Synchronous precursor selection (SPS) was enabled at all times to include up to 5 MS2 fragment ions in the MS3 scan.

Raw mass spectrometry data were analyzed with the MaxQuant software suite ([22]; version 1.6.4.0) as described previously [102]. A false-discovery rate of 0.01 for proteins and peptides and a minimum peptide length of 7 amino acids were set. TMT tags on peptide *N*-termini and lysine residues (+ 229.162932 Da) and carbamidomethylation of cysteine residues (+ 57.02146 Da) were set as static modifications, whereas methionine oxidation (+ 15.99492 Da) was set as variable modification. The Andromeda search engine was used to search the MS/MS spectra against the Uniprot database (taxonomy: *Homo sapiens*, release January 2019) concatenated with the reversed versions of all sequences. The enzyme specificity was set to trypsin and a maximum of two missed cleavages was allowed. The FDR for both peptides and proteins was set to 0.01. The peptide tolerance was set to 10 ppm, the fragment ion tolerance was set to 0.6 Da for CID spectra and 20 ppm for MS3 reporter ion spectra. MaxQuant automatically quantified peptides based on the ‘reporter ion MS3 setting’. Before further statistical analysis, known contaminants and reverse hits were removed. Reporter ion intensities were adjusted to correct for the isotopic impurities of the different TMT reagents (according to the manufacturer’s specifications). For further analysis, we used the R packages vsn and limma [48, 97].

Differentially expressed proteins were selected based on the following thresholds: logFC <1, FDR < 0.05 (Suppl. Table 5). For further analysis, the ALSP proteomics data set was compared to published gene sets to find overlapping proteins, including human microglia [38] and astrocytic genes [126], reactive astrocyte-associated genes [35,126] and extracellular matrix-related genes [77] (Suppl. Table 6). For the comparison to published gene sets, the thresholds for LogFC was set to < − 0.3 and > 0.3, with FDR < 0.05 to not exclude less expressed proteins and pick up subtle changes.

### Image acquisition

Confocal imaging was performed using a Leica SP5 intravital imaging setup with a 40x/1.3 NA oil objective for stained sections imaging, with 405, 488 and 633 nm lasers. Z‐stack images (z step size 0.5–1 µm) were acquired for all experiments. Brightfield images of the HRP stained sections were obtained using an Olympus DP72 light microscope or a Leica DM600B microscope (Leica microsystems). Sections were placed under the microscope and the experimenter determined whether the region visible was in grey or white matter. If no artifacts and/or tissue damage were present, the image was acquired on that location (5–10 images per region). We recorded representative images of the microglia population, and hence, microglial clusters were included in the quantification (1–2 images), since these clusters are typical for ALSP neuropathology.

### Quantification and statistical analysis

Images were processed and quantified using the Fiji image processing package [101]. The number of microglia and astrocytes were counted blindly and manually with an even ROI counting surface between ALSP and controls. Morphology of astrocytes was analyzed using the Sholl plugin in ImageJ [36]. Statistical significance of the Sholl analysis was determined by one- or two-way ANOVA based on the mean area under the curve (AUC). Intracellular LAMP1 staining quantification was done as follows: based on the astrocyte marker channel, an ROI was generated using a standardized threshold to analyze LAMP1 + puncta within single cells. This was overlaid in the corresponding LAMP1 channel, and only the astrocyte ROI was kept using the Clear Outside tool. Intracellular particles were then analyzed on a binary image (thresholded) with the Analyze Particles tool. Statistical analysis was performed using GraphPad Prism 8, including (nested) one-way ANOVA with Bonferroni multiple testing correction. Data are presented as mean ± SD, as indicated. A p value < 0.05 was considered significant.

## Zebrafish models

### CRISPR-Cas9 genome editing in zebrafish

#### Design of ssDNA oligo to generate ALSP-pathogenic variants into the zebrafish genome

To introduce pathogenic CSF1R missense variants causing ALSP into the zebrafish genome, we used CRISPR/Cas9 with co-injection of an ssDNA oligo [Integrated DNA Technologies (IDT), standard desalted oligo (STD)] [115]. This ssDNA oligo, GCATCCACCGAGACGTGGCaGtCAGAAACG, contained 20 bp homology arms up- and downstream of the double-stranded break created by Cas9, a missense variant causing the ALSP-pathogenic amino acid substitution (Ala784Val) and a mutation in the PAM site without changing an amino acid, to avoid re-cutting by Cas9. The mutations generated a restriction site (DrdI), which was used for genotyping.

### Cas9/gRNA complex and ssDNA oligo injections into zebrafish larvae

The Alt-R™ CRISPR-Cas9 System of IDT was used to generate a gRNA with target sequence TGCATCCACCGAGACGTGG. Equal amounts of crRNA (crispr RNA) and tracrRNA (trans-activating crispr RNA) in Duplex Buffer (IDT) were incubated for 5 min at 95 °C followed by cooling to RT to generate 50 µM gRNA duplex. The SP-Cas9 plasmid (Addgene plasmid #62731) used for the production of Cas9 protein was deposited by Niels Geijsen [28]. Cas9 nuclease was synthetized as described [28]. 50 pmol gRNA was mixed with 4 ng Cas9 protein to form gRNA-Cas9 RNPs. Next, 30 pmol oligo and 0.4 µl of 0.5% Phenol Red was added in a total volume of 6 µl. 1 nl was injected in the zebrafish embryo at the one-cell stage, grown up and used in a founder screen.

### Founder screen to generate patient-specific missense mutant and out-frame deletion mutant

Injected embryos were raised up and crossed out against wild-type (WT) fish. Twenty-four embryos per founder fish were lysed and genotyped by PCR and digestion (see ‘Genotyping of zebrafish and zebrafish larvae’). PCR products containing a restriction site were sequenced by Sanger sequencing. Siblings were raised up and genotyped by PCR and Sanger sequencing. Zebrafish with the ALSP-causing mutation (*csf1ra*^*A784V*/+^) and zebrafish with a 5 bp deletion (p.(Val782Glyfs*16), csf1ra ^ex17Δ5/+^) were selected and crossed to obtain a stable mutant line.

### Genotyping of zebrafish and zebrafish larvae

Adult zebrafish were anesthetized with 0.016% MS-222 and a small piece of the caudal fin was cut and lysed in 80 µl 50 mM KOH. Zebrafish larvae were euthanized and placed in single tubes containing 80 µl 50 mM KOH per larva. They were incubated at 95 °C for 30 min. 8 µl 1 M Tris–HCl pH 8 was added, and 1 µl of the lysate was used for PCR (TouchDownPCR: 5′ 95 °C, 30″ 95 °C, from 65 °C to 55 °C in 10 cycles, 45″ 72 °C, 30″ 93 °C, 30″ 58 °C, 45″ 72 °C repeated 25 times, 3′ 72 °C). For digestion, 5 µl of PCR product was digested in a mix of 20 µl, incubated for 1 h, and loaded on a 2.2% Tris-borate-EDTA (TBE) agarose gel. See Table 1 for the primer sequences.

**Table 1:** Primer sequences for genotyping

Allele	F	R
Csf1ra V614M	CAAAGCACTTCATGGGACCT	CATTACCACACCGACACAGC
	Mut-For: AAGAGGACAACATCACACGAA	
	Wt-For: AAGAGGACAACATCACACGAG	
Csf1ra A784V	GGAGTGTCAGGAGGACTCG	CTGATCAAAGGGAAGGTTGC
	Digestion with DrdI. DrdI cuts in mutant allele. Wt allele: 410bp. Mutant allele: 212bp, 198bp	
Csf1ra ex17Δ5	GGAGTGTCAGGAGGACTCG	CTGATCAAAGGGAAGGTTGC
	Digestion with BmgBI. BmgBI cuts in wt allele. Wt allele: 212bp, 198bp. Mutant allele: 405bp	
Csf1rb^−/−^	CTTGCTGACAAATCCAGCAG	GAGCTAACCGGACAAACTGG
	Digestion with MspI. MspI cuts in the wt allele. Wt allele: 203bp, 132bp, 71bp. Mutant allele: 330bp, 71bp	

### Neutral red staining and imaging

Neutral red (NR) microglial staining was performed as previously described [63]. Briefly, 3 or 5 dpf larvae were incubated in E3 medium containing NR (Sigma-Aldrich) (2.5 µg/ml) and 0.003% PTU for 2 h at 28 °C, after which they were rinsed with E3 medium containing 0.003% PTU for 30 min at 28 °C. Larvae were anesthetized with 0.016% MS-222 and mounted dorsal side up in 1.8% low-melting-point agarose in E3 medium. Serial images (2–4) in the z-plane were acquired with a Leica M165 FC microscope using a 10 × dry objective and a Leica DFC550 camera. The serial images were stacked using Fiji and the NR^+^ microglia were manually counted in the midbrain, since microglia first colonize the optic tectum in the midbrain around 2 dpf [46]. Since all experiments were done on incrosses of mutants, image acquisition and counting by two independent researchers was performed blindly.

### Quantitative PCR of *csf1ra* in zebrafish larvae

Total RNA of 20 zebrafish larvae (5 dpf) per sample was isolated using TRIzol™ Reagent (ThermoFisher Scientific) and cDNA prepared using iSCRIPT cDNA Synthesis Kit (BioRad). qPCR was performed, with biological duplo’s, using iTaq universal SYBR Green Supermix in a CFX96RTS thermal cycler, in triplo (Bio-Rad). Relative gene expression was determined following the ΔΔct method. The following forward/reverse primer pairs were used:

*csf1ra* F: ATGACCATACCCAACTTTCC/R: AGTTTGTTGGTCTGGATGTG.

*mpeg1.1* F: CCCACCAAGTGAAAGAGG/R: GTGTTTGATTGTTTTCAATGG.

*eef1a1* F: CATTGCTCTCTGGAAATTCG/R: CACAGTCAGCCTGAGAAGTACC.

### LysoTracker red staining

Zebrafish larvae (*n* =  ~ 12) were incubated in 2 ml tubes with 10 μM LysoTracker™ Red DND-99 (1:100) (ThermoFisher, Waltham, MA) in 200 μl E3/PTU. Tubes were kept in the dark at 28 °C for 40 min with the lids open. Afterwards, larvae were washed with E3/PTU for 10–15 min in the dark at 28 °C. Imaging started within 45 min after staining, to preserve the LysoTracker signal as best as possible.

### In situ hybridization

Whole-mount in situ hybridization was performed as previously described [116]. Riboprobes were prepared via T7 transcription using digoxigenin-labeled NTPs (Roche, Mannheim, Germany) and a cDNA template as described previously [14]. Primers are listed as follows, with the underlined region indicating the T7 promoter sequence incorporated into the reverse primer: *mbpa:*

F CTAAGTCGAGGGGAGAAAGCC/R: TAATACGACTCACTATAGGGAGGGCATACAATCCAAGCCA (product size 885 bp).

*plp1b*:

F TCCTCTATGGACTGTTGCTGCTG/R TAATACGACTCACTATAGGGACAATCACACACAGGAGGACCAA (product size 1052 bp).

### Intracerebral microinjection in larvae

Red pHrodo-labeled myelin was gifted by Inge Huitinga (Amsterdam UMC, The Netherlands Brain Bank). Briefly, myelin was isolated from post-mortem brain tissue of a pool of healthy control donors, and labeled with the pH-sensitive fluorescent dye pHrodo, as previously described [44]. Five nanogram of pHrodo-labeled myelin (5 nl injection of stock: 1 mg pHrodo-myelin/ml) was injected intra-cerebrally in 3 dpf larvae to quantify myelin uptake in lysosomes by astrocytes. Three hours post-injection (hpi), larvae were imaged to assess the pHrodo myelin signal. Eventually, we obtained in vivo time-lapse imaging data from 18 hpi until 21 hpi.

### In vivo confocal image acquisition

Zebrafish were anesthetized using 0.016% MS-222 and mounted in 1.8% low-melting-point agarose in E3 medium. The imaging dish was covered with E3 medium containing MS-222 during imaging. Larvae were imaged on a Leica SP5 intravital microscope using a 20 × water dipping objective (Leica Plan-Apochromat, NA = 1.0) using 488 nm and 561 nm lasers. Confocal z-stack (step size 0.5–3 µm) images were acquired in vivo. For in vivo time-lapse imaging after pHrodo myelin injection, images (z-stack around 10, step size 2 µm) were acquired every 3 min for 3 h (18–21 hpi).

### Locomotor activity assays

To assess the locomotor activity of zebrafish larvae (5 dpf), a locomotor activity assay was performed using an infrared camera system (DanioVision™ Observation chamber, Noldus) and EthoVision^®^ XT software (Noldus) as previously described [61]. WT, *csf1ra*^V614M/+^ and *csf1ra*^V614M/V614M^ larvae (*n* = 16/group), in 48-well plates, were subjected to a light/dark routine. The dawn routine, 15-min habituation in the dark (0% light intensity), followed by the routine described in Table 2, comprised 3 h 15 min. This experiment was performed twice. Distance traveled (mm) per second was measured.

**Table 2:** Dusk–dawn routine

Light intensity (%)	0	0– > 50	50	50– > 0	0	0– > 50	50	50– > 0	0
Duration (min)	15	15	30	15	30	15	30	15	30

### Quantification and statistical testing

Images were processed and quantified using Fiji. NR + microglia were counted blindly, by two independent researchers, as the genotype of the larvae was not known yet because of the heterozygous incross. Oligodendrocyte lineage cells were counted blindly and manually using a maximum projection of the Z-stack. Myelinated area was analyzed on a maximum projection of a Z-stack, based on a binary image (threshold) and set ROI similar for all fish. LysoTracker inclusions within astroglia were counted blinded and manually through the whole Z-stack per 2 Z’s. Statistical analysis was performed using GraphPad Prism 8, including (nested) one-way ANOVA with Bonferroni multiple testing correction. Data are presented as mean ± SD. A *p* value < 0.05 was considered significant.

## Results

### ALSP patients show an overall loss of (homeostatic) microglia and altered microglial distribution

Recently, we and others showed reduced density of IBA1 + microglia in various brain areas in post-mortem tissue of ALSP patients, suggesting that microglial depletion could be a general feature of ALSP [83, 85, 113]. However, it is still unclear whether microglial depletion is a key hallmark and initial pathological event in ALSP. We had the unique opportunity to explore microglia in a larger group of late-stage patients and an intermediate disease stage by examining post-mortem frontal gyrus tissue of two ALSP patients who died relatively early in the disease. A summary of the patient histories is provided in the Methods section and in Supplementary Table 1, Online Resources. MRI showed bilateral white matter lesions, corpus callosum involvement and mild generalized brain atrophy in both patients, and restricted diffusion in the majority of the lesions in intermediate-stage patient 1 (Fig. 1a). In intermediate-stage patient 1, the parieto-occipital white matter is predominantly affected, in intermediate-stage patient 2, the frontal white matter (Fig. 1a). We performed immunohistochemical (IHC) analyses using post-mortem brain tissue of the frontal gyrus of intermediate-stage ALSP patients (*n* = 2), late-stage ALSP patients (*n* = 6) and controls (*n* = 3) (Suppl. Table 1, Online Resources) [69]. All ALSP brain tissue samples showed the typical hallmarks of ALSP, including axonal spheroids, pigmented glia and loss of myelin sheaths and white matter degeneration (Suppl. Figure 1a, b, c, Online Resources).

Next, we quantified homeostatic microglia based on the markers TMEM119+ and P2RY12+ , both exclusively expressed by microglia [10, 43]. In intermediate-stage patients, there were significantly fewer TMEM119 + microglia in white matter, but not in grey matter (Fig. 1b, d). By contrast, P2RY12+ microglia were significantly reduced in both grey and white matter (Fig. 1c, e). Intermediate-stage ALSP tissue also showed a clustered distribution of microglia predominantly in white matter (Fig. 1f). In late-stage ALSP grey and white matter, fewer TMEM119+ and P2RY12+ microglia were present than in control tissue (Fig. 1b–e). In ALSP, TMEM119+ and P2RY12+ microglia presented in a clustered distribution, similar to what we have observed before [83] (Fig. 1f). Particularly in white matter, some areas were almost devoid of microglia while others showed large distinct microglial clusters (Fig. 1f), with abundant amoeboid CD163+ cells, indicating actively phagocytosing microglia or macrophages, possibly originating from the circulation (Fig. 1g, Suppl. Fig. 1d, Online Resources). Intermediate-stage ALSP white matter also displayed clusters of CD163+ cells throughout the frontal gyrus, which had a more ramified morphology than in late-stage ALSP, although amoeboid CD163+ cells were also abundant (Suppl. Fig. 1d, e, Online Resources). Altogether, this further indicates that ALSP patients exhibit an overall loss of (homeostatic) microglia and clustered microglial distribution—predominantly in white matter—which is already present in relatively early disease stages.

### Heterozygous pathogenic missense variants in *csf1ra* result in microglial depletion in early development

**Fig. 2:**
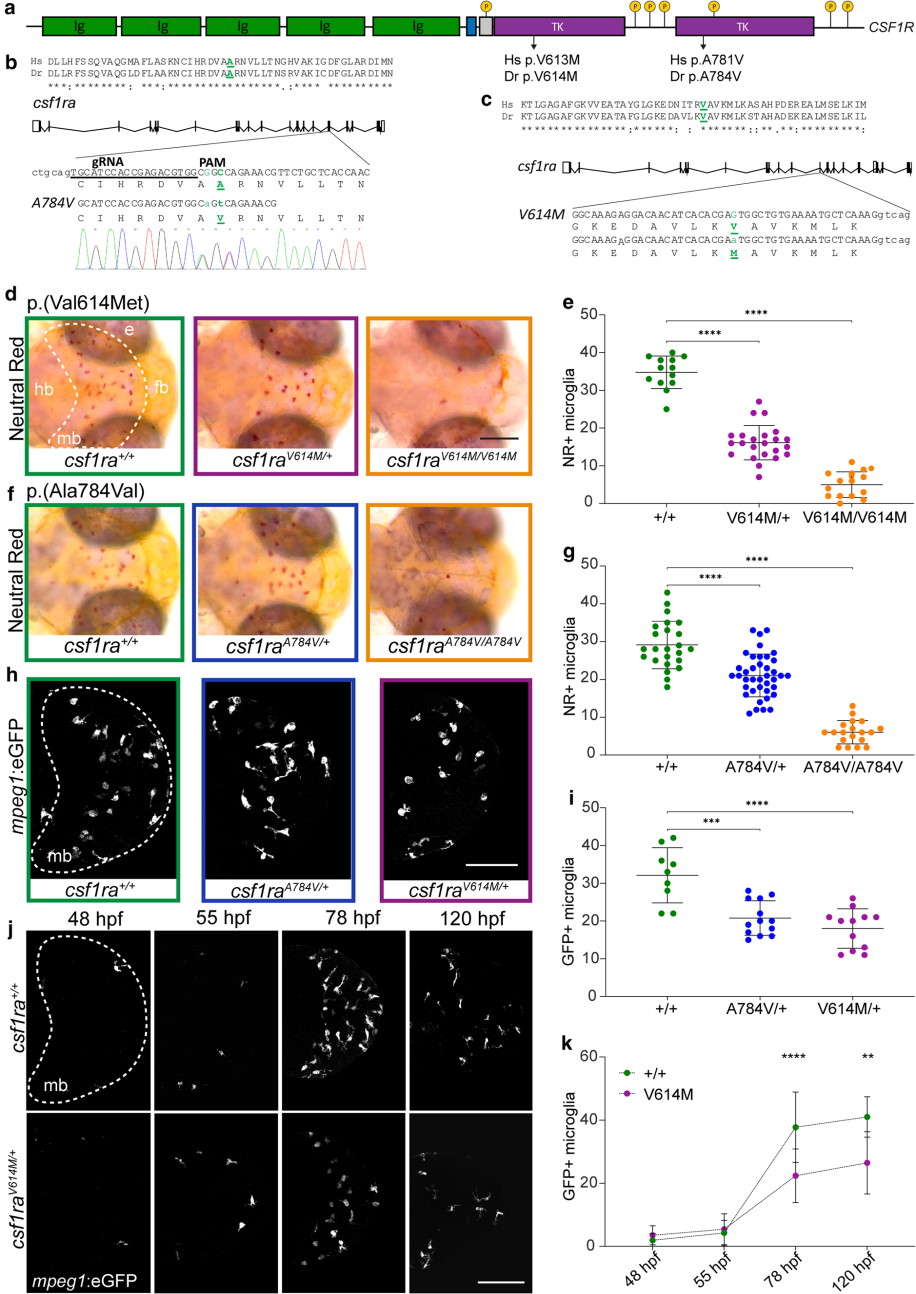
Heterozygous pathogenic missense variants in *csf1ra* result in microglial depletion in early development. **a** Schematic representation of the *CSF1R* gene, with five transmembrane immunoglobulin (ig) domains and two intracellular tyrosine kinase domains (TKD). Locations of the missense variants (human (Hs) and zebrafish (Dr)) are depicted, as well as tyrosine phosphorylation sites (p). **b** Schematic representation of *csf1ra* with: the location of the Ala784Val missense variant, located in exon 17 in the second TKD, the 19 bp gRNA, the PAM motif and the co-injected 30 bp oligo containing the missense variant, resulting in an Ala to Val change. **c** Schematic representation of *csf1ra* and the Val614Met missense variant in exon 13 [87]. **d** Representative images of the midbrain (mb, dashed line) after NR staining of *csf1ra*^+/+^ (green), *csf1ra*^V614M*/*+^ (purple) and *csf1ra*^V614M*/*V614M^ (orange) larvae at 3 dpf. **e** Quantification of NR + microglia in *csf1ra*^+/+^ (green, *n* = 13), *csf1ra*^V614M*/*+^ (purple, *n* = 23) and *csf1ra*^V614M*/*V614M^ (orange, *n* = 16) larvae at 3 dpf in the midbrain. **f** Representative images of the midbrain after NR staining of *csf1ra*^+/+^ (green), *csf1ra*^A784V*/*+^ (blue) and *csf1ra*^A784V*/*A784V^ (orange) larvae at 3 dpf. **g** Quantification of NR + microglia in *csf1ra*^+/+^ (green, *n* = 24), *csf1ra*^A784V*/*+^ (blue, *n* = 39) and *csf1ra*^A784V*/*A784V^ (orange, *n* = 19) larvae at 3 dpf. **h** Representative images of *mpeg1*:GFP + microglia in the midbrain (dashed line, mb) of *csf1ra*^+/+^ (green), *csf1ra*^A784V*/*+^ (blue) and *csf1ra*^V614M*/*+^ (purple) larvae at 3 dpf. **i** Quantification of *mpeg1*:GFP + microglia in *csf1ra*^+/+^ (green, *n* = 9), *csf1ra*^A784V*/*+^ (blue, *n* = 13) and *csf1ra*^V614M*/*+^ (purple, *n* = 12) at 3 dpf. **j** Representative longitudinal images of *mpeg1*:GFP + microglia in the midbrain (dashed line, mb) of the same *csf1ra*^+/+^ and *csf1ra*^V614M*/*+^ larvae from 48 to 120 hpf. **k** Quantification of *mpeg1*:GFP + microglia in the midbrain of *csf1ra*^+/+^ (*n* = 6) and *csf1ra*^V614M*/*+^ (*n* = 9) larvae from 48 to 120 hpf. Days-post-fertilization (dpf), eye (e), forebrain (fb), hindbrain (hb), midbrain (mb), neutral red (NR). One-way or two-way ANOVA test was preformed to test for significance (*p* < 0.05). Error bars represent SD. *p < 0.05, **p < 0.01, ***p < 0.001 ****p < 0.0001. Scale bar equals 100 μm (**h**, **j**) and 200 μm (**d**)

As brain tissue of ALSP patients preceding disease onset is not accessible, we investigated the effect of pathogenic CSF1R missense variants in zebrafish brain development in vivo. Zebrafish have two *CSF1R* homologs, *csf1ra* and *csf1rb*, and we and others previously showed that *csf1ra* is particularly important for microglia during brain development [37, 46, 62, 81]. First, we examined zebrafish larvae with the missense variant p.(Val614Met) in Csf1ra in a *csf1rb-*deficient background to prevent genetic compensation (Fig. 2a, c) [83, 87]. Val614Met corresponds to p.Val613 in hCSF1R, a highly conserved residue in TKD1 where many ALSP-causing variants are located (Suppl. Table 2, Online Resources). To date, no missense variants have been reported at residue 613 in gnomAD (v 2.1.1) in the healthy population, although the missense variant p.(Val613Leu) was identified in an ALSP patient [20], making p.(Val613Met) a pathogenic *CSF1R* variant. Zebrafish microglia colonize the embryonic optic tecta in the midbrain around 2 days post-fertilization (dpf) and stain positive for neutral red (NR) and the transgenic marker *mpeg1*-*GFP* [46]. We, therefore, quantified NR-stained and transgenically labeled *mpeg1:*GFP + microglia in the midbrain in progeny of incrossed *csf1ra*^V614M*/*+^ zebrafish. We found > 50% fewer microglia at 3 and 5 dpf in *csf1ra*^V614M*/*+^ zebrafish, whereas homozygous *csf1ra*^V614M*/*V614M^ mutants, in line with previous studies, had only a few remaining microglia (Fig. 2d, e, h, i) [37, 83].

Most ALSP-causing CSF1R variants are located in TKD2 (Suppl. Table 2, Online Resources). We, therefore, edited the *csf1ra* genomic locus using a CRISPR/Cas9 knock-in strategy in a *csf1rb*-deficient background and introduced p.(Ala784Val) (Fig. 2a, b). Ala781Val (Ala784Val in zebrafish) occurs at a highly conserved residue and several variants at this position have been described in ALSP patients [2, 21, 93, 117] (Suppl. Table 2, Online Resources). We incrossed heterozygous *csf1r*^A784V*/*+^ zebrafish and observed ~ 30% fewer NR + and *mpeg1:*GFP + microglia as early as 3 dpf, whereas *csf1ra*^A784V/A784V^ mutants had almost no microglia (Fig. 2f, g, h, i).

To define the time point from which reduced microglia can be detected, we performed longitudinal analyses, quantifying *mpeg1:*GFP + microglia at 48, 55, 78 and 120 hpf in the same individual *csf1ra*^*V614M/*+^ and control larvae (Fig. 2j, k). At 48 and 55 hpf, the number of *mpeg1:*GFP + microglia did not differ between *csf1ra*^*V614M/*+^ and controls, but from 78 hpf *csf1ra*^*V614M/*+^ mutants showed fewer *mpeg1:*GFP + microglia (Fig. 2j, k). Altogether, these data indicate that ALSP-causing CSF1R missense variants in zebrafish cause a 30–50% reduction of microglia as early as day 3 of embryonic development.

### Heterozygous ALSP-causing CSF1R missense variants act dominant negatively, and differentially, in decreasing the number of microglia

**Fig. 3:**
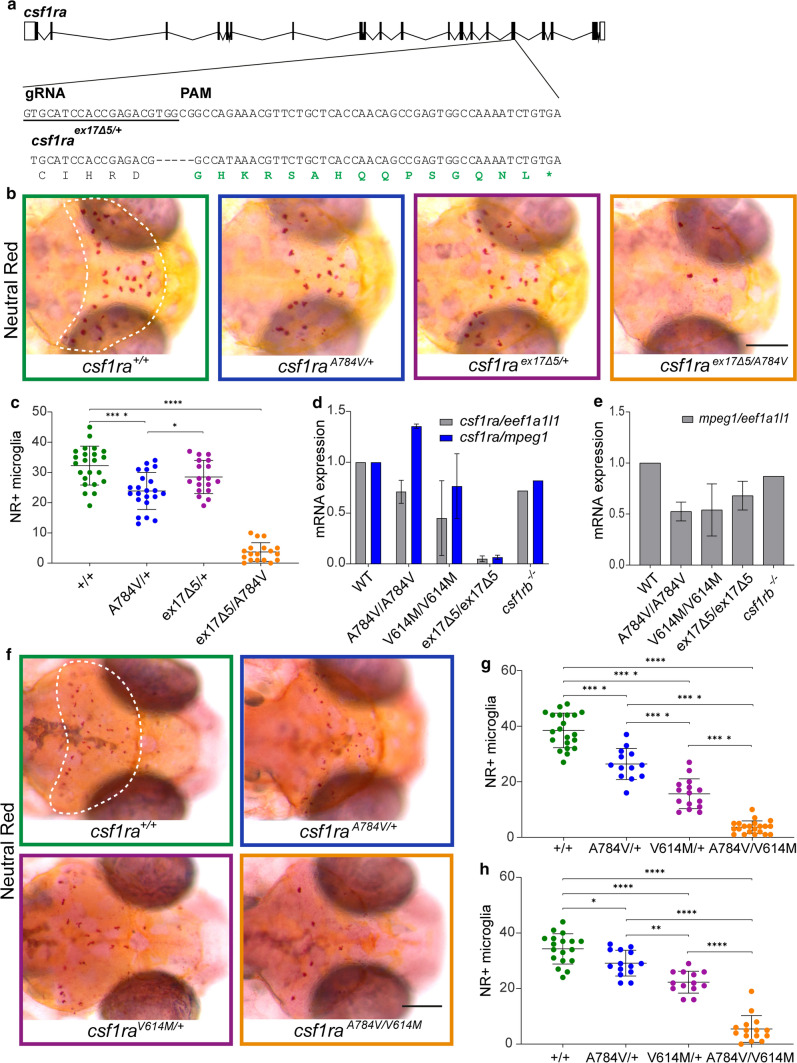
Heterozygous ALSP-causing CSF1R missense variants act dominant negatively in reducing the number of microglia. **a** Schematic representation of *csf1ra* with the 20 bp gRNA and the PAM motif, and 5 base pair (bp) deletion in exon 17 indicated, resulting in a frameshift (green) followed by a premature stop codon. **b** Representative images of the midbrain (dashed line) after NR staining of progeny of *csf1ra*^A784V*/*+^ crossed with *csf1ra*^ex17∆5/+^ at 3 dpf. **c** Quantification of NR + microglia in the midbrain of *csf1ra*^+/+^ (green, *n* = 23), *csf1ra*^A784V*/*+^ (blue, *n* = 22), *csf1ra*^ex17∆5/+^ (purple, *n* = 18) and *csf1ra*^ex17∆5/A784V^ (orange, *n* = 18). **d** Expression of *csf1ra* mRNA in WT, *csf1ra*^A784V*/*A784V^, *csf1ra*^V614M*/*V614M^ and *csf1ra*^+/+^ (*n* = 20/group) normalized to expression of *eef1a1l1* (grey) and microglial gene *mpeg1* (blue). **e** Expression of *mpeg1* mRNA normalized to expression of housekeeping gene *eef1a1l1* in WT, *csf1ra*^A784V*/*A784V^, *csf1ra*^V614M*/*V614M^ and *csf1ra*^+/+^. **f** Representative images of the midbrain (dashed line) after NR staining of progeny of *csf1ra*^A784V*/*+^ crossed with *csf1ra*^V614M*/*+^ at 3 dpf. **g, h** Quantification of NR + microglia in the midbrain at 3 dpf (**g**) and 5 dpf (**h**) in *csf1ra*^+/+^ (green, *n* = 21, 18), *csf1ra*^A784V*/*+^ (blue, *n* = 13, 14), *csf1ra*^V614M*/*+^ (purple, *n* = 15, 13) and *csf1ra*^A784V*/*V614M^ (orange, *n* = 22, 15). Days-post-fertilization (dpf), neutral red (NR), wild type (WT). One-way ANOVA test was preformed to test for significance (*p* < 0.05). Error bars represent SD. *p < 0.05, ****p < 0.0001. Scale bars equal 200 μm

Several parents of patients with BANDDOS due to bi-allelic *CSF1R* variants carry a heterozygous variant resulting in a frameshift, which is expected to cause a complete loss of function of one *CSF1R* allele, but do not show signs of ALSP or neurological dysfunction [74, 81]. To test whether a heterozygous null allele would lead to altered numbers of microglia, we generated a frameshift variant in exon 17 (p.Val782Glyfs*16)—in the same genomic region as the Ala784Val variant—with a 5 bp deletion causing a frameshift and premature stop, in a *csf1rb*-deficient background. To compare these variants, we crossed *csf1ra*^ex17∆5/+^ with *csf1ra*^A784V/+^ mutants to limit potential effects of experimental or genetic background differences (Fig. 3a). At 3 dpf, *csf1ra*^ex17∆5/+^ zebrafish showed normal numbers of microglia, whereas *csf1ra*^A784V/+^ larvae showed fewer microglia than control and *csf1ra*^ex17∆5/+^-mutant siblings (Fig. 3b, c). By contrast, *csf1ra*^ex17∆5/A784V^ larvae had almost no microglia, indicating that both variants are loss of function (Fig. 3b, c).

Heterozygous frameshift variants can lead to mono-allelic expression of CSF1R due to NMD, consequently leading to ~ 50% lower CSF1R activity. By contrast, missense variants may lead to deficient receptor homo- and heterodimers, which would result in around 75% loss of function [92]. We tested expression of *csf1ra* in *csf1ra*^ex17∆5/ex17∆5^ zebrafish larvae and homozygous missense mutants by qPCR, revealing strongly reduced expression in *csf1ra*^ex17∆5/ex17∆5^ mutants, but not in missense mutants, compared to controls, even when expression was normalized to *mpeg1* to account for microglial depletion (Fig. 3d, e). This shows that the exon 17 frameshift variant results in NMD of *csf1ra*, which, together with our finding that the variant does not result in a loss of microglia, supports the conclusion that ALSP-causing CSF1R missense variants act dominant negatively.

It is possible that individual ALSP-causing CSF1R missense variants have differential effects on receptor activity and microglial function and numbers. To discern whether the p.(Ala781Val) and p.(Val613Met) variants would differ in their effect on microglia, we incrossed *csf1r*^A784V/+^ and *csf1ra*^V614M/+^ zebrafish to reduce potential genetically or experimentally induced effects and quantified microglia in their progeny. Both *csf1ra*^A784V/+^ larvae and *csf1ra*^V614M/+^ larvae consistently showed reduced numbers of microglia at 3 and 5 dpf, whereas *csf1ra*^A784V/V614M^ mutants showed almost no microglia (Fig. 3f, g, h). However, we also found, consistent with analyses in the individual mutants, that *csf1ra*^V614M/+^ mutants had slightly fewer microglia than *csf1ra*^A784V/+^ mutants (Fig. 3f, g, h). Thus, pathogenic CSF1R missense variants could have differential effects on the number of microglia. Altogether, ALSP-causing CSF1R missense variants, but not frameshift null variants, act dominantly and cause strongly reduced numbers of microglia, whereas *csf1ra* haploinsufficiency is insufficient to cause microglial depletion.

### Heterozygous pathogenic CSF1R missense variants cause defective proliferation of microglia

To investigate what underlies the reduced numbers of microglia, we performed in vivo imaging of *csf1ra*^*A784V/*+^, *csf1ra*^*V614M/*+^ and *csf1ra*^+/+^ (*csf1ra* wild-type) larvae at 24 hpf and from 32 to 48 hpf, when yolk sac macrophages (YSM) start to colonize the brain [45,46] (Suppl. Fig. 2a, c, Online Resources). YSM numbers in mutants were normal at 24 hpf and between 32 and 48 hpf, and were able to colonize the embryonic brain (Suppl. Fig. 2a–d, Online Resources). From 54 to 72 hpf, we observed a considerably lower frequency of microglial proliferation in *csf1ra*^*V614M/*+^ mutants than in *csf1ra*^+/+^ larvae in the midbrain (Suppl. Fig. 2e, f, Online Resources). Altogether, there is no obvious difference in YSMs or their migration into the brain in heterozygous mutants, whereas microglia in heterozygous missense mutants show a lower proliferation frequency.

### Homozygous missense *csf1ra* mutants show abnormalities in myelination and behavior

**Fig. 4:**
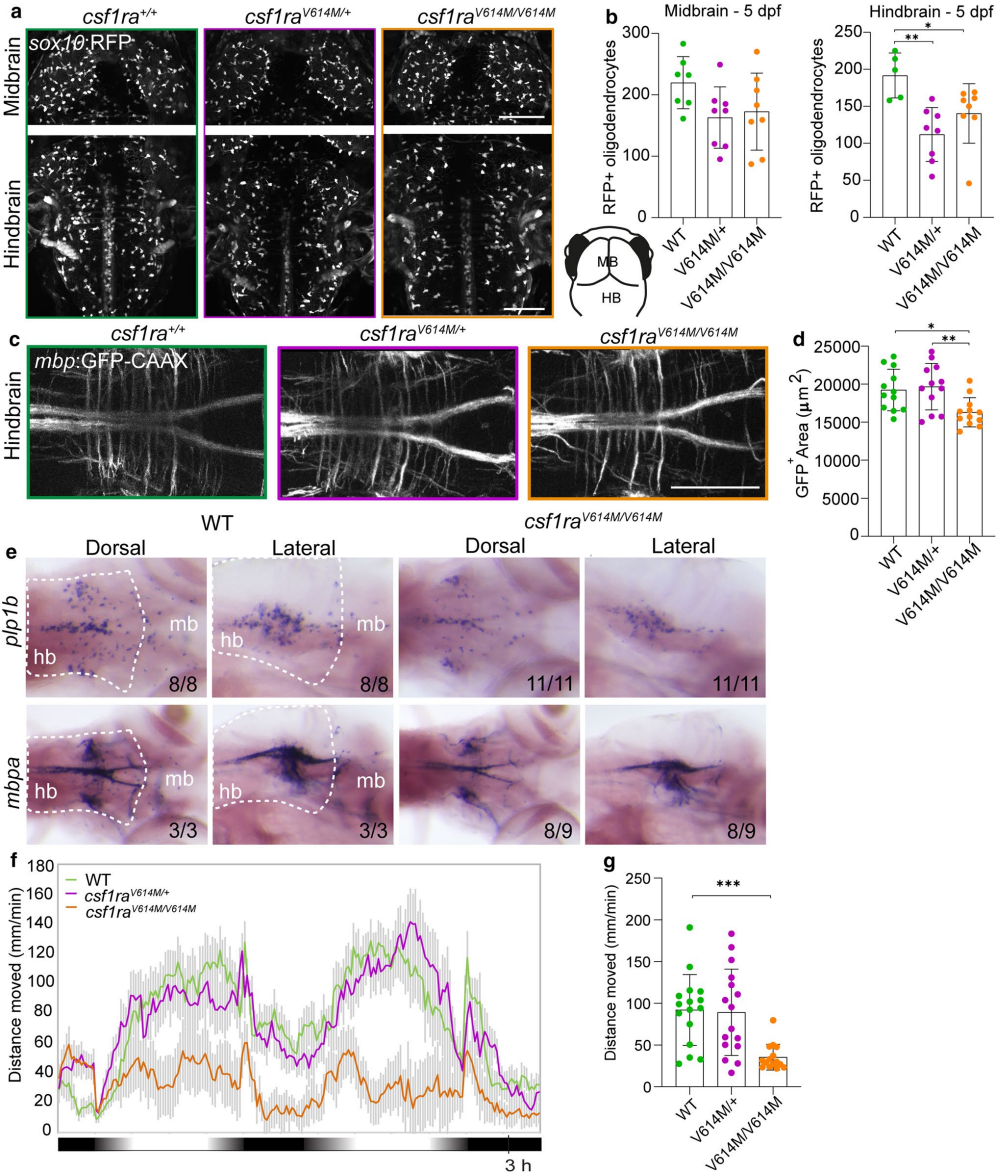
Homozygous missense variants in *csf1ra* causes myelin and behavioral abnormalities related to leukodystrophy. **a** Representative images of the midbrain (top) and the hindbrain (bottom) of *csf1ra*^+/+^ (green), *csf1ra*^V614M*/*+^ (purple) and *csf1ra*^V614M*/*V614M^ (orange) larvae in a *sox10*:RFP background, visualizing oligodendrocytes, at 5 dpf. Lower right: schematic of zebrafish embryonic midbrain (mb) and hindbrain (hb). **b** Quantification of the number of *sox10*:RFP + oligodendrocytes in the midbrain and hindbrain at 5 dpf in WT (green), *csf1ra*^V614M*/*+^ (purple) and *csf1ra*^V614M*/*V614M^ (orange) larvae. **c** Representative images of the hindbrain of WT (green), *csf1ra*^V614M*/*+^ (purple) and *csf1ra*^V614M*/*V614M^ (orange) larvae in a *mbp*:GFP-CAAX background, visualizing myelin sheaths, at 5 dpf. **d** Quantification of the total *mbp*:GFP + myelinated area (μm^2^) in the hindbrain at 5 dpf in WT (green), *csf1ra*^V614M*/*+^ (purple) and *csf1ra*^V614M*/*V614M^ (orange) larvae. **e** Representative images of in situ hybridization of *plp1b* (top) and *mbpa* (bottom) in WT (left) and *csf1ra*^V614M*/*V614M^ (right) larvae at 5 dpf, showing reduce number of *plp1b* + mature myelinating oligodendrocytes (11/11 larvae) and reduced *mbpa* + myelin sheaths and myelinating oligodendrocytes (8/9 larvae) in *csf1ra*^V614M*/*V614M^ larvae. Dashed lines show the hindbrain (hb). **f** Representative graph showing the total distance traveled (mm) by larvae per 1 min during the dusk–dawn routine (total time: 3 h 15 min), of *csf1ra* mutants in a *csf1rb-*deficient background. Grey shading shows the standard error of the mean (SEM). **g** Quantification of the total distance moved throughout the experiment excluding the dark period. *n* = 16 larvae per genotype. Days-post-fertilization (dpf), hindbrain (hb), midbrain (mb). One-way ANOVA test was preformed to test for significance (*p* < 0.05). Error bars represent SD, unless stated otherwise. *p < 0.05, **p < 0.01 ***p < 0.001. Scale bars equal 100 μm

Progressive white matter lesions are a major hallmark of ALSP. To study whether *csf1ra*-mutant zebrafish have myelin abnormalities, we performed in vivo imaging of *csf1r*^*WT*^, *csf1ra*^*V614M/*+^ and *csf1ra*^*V614M/V614M*^ larvae in *sox10*:RFP and *mbp*:GFP-CAAX transgenic backgrounds, to visualize oligodendrocytes and myelin sheaths, respectively [27, 56]. At 3 dpf, both heterozygous and homozygous larvae had fewer *sox10*:RFP + oligodendrocytes in the mid- and hindbrain than controls, but not in the spinal cord (Suppl. Fig. 3a–e, Online Resources). At 5 dpf, both mutant groups showed fewer *sox10*:RFP + oligodendrocytes in the hindbrain, but not in the midbrain nor the spinal cord (Fig. 4a, b, Suppl. Fig. 3a–e, Online Resources). We wondered whether the decrease in hindbrain *sox10*:RFP + oligodendrocytes in the mutants reflected impaired myelination. In *csf1ra*^*V614M/V614M*^ larvae, but not in *csf1ra*^*V614M/*+^, *mbp:*GFP + myelinated area was smaller than in WT controls (Fig. 4c, d). In situ hybridization (ISH) with probes for *mbpa* and *plp1b* validated the decrease in the number of mature myelinating oligodendrocytes and myelin content in *csf1ra*^*V614M/V614M*^ larvae at 5 dpf (Fig. 4 e). As motor decline is prevalent in ALSP patients, we measured locomotor activity of control and missense mutant larvae during a dusk–dawn behavioral assay as described previously [61] (Fig. 4f), and found that only homozygous mutant larvae had reduced locomotor activity (Fig. 4g). Together, both *csf1ra*^*V614M/*+^ and *csf1ra*^*V614M/V614M*^ larvae have an early reduction of *sox10*:RFP + oligodendrocyte cell numbers in the hindbrain, but only in *csf1ra*^*V614M/V614M*^ mutants this was accompanied by a locomotor phenotype and smaller *mbp*:GFP + area, indicating marginally reduced myelination in the hindbrain.

### Transcriptome and proteome analysis of relatively spared tissue reveals increased stress response and astrocyte-related proteins in ALSP patients

**Fig. 5:**
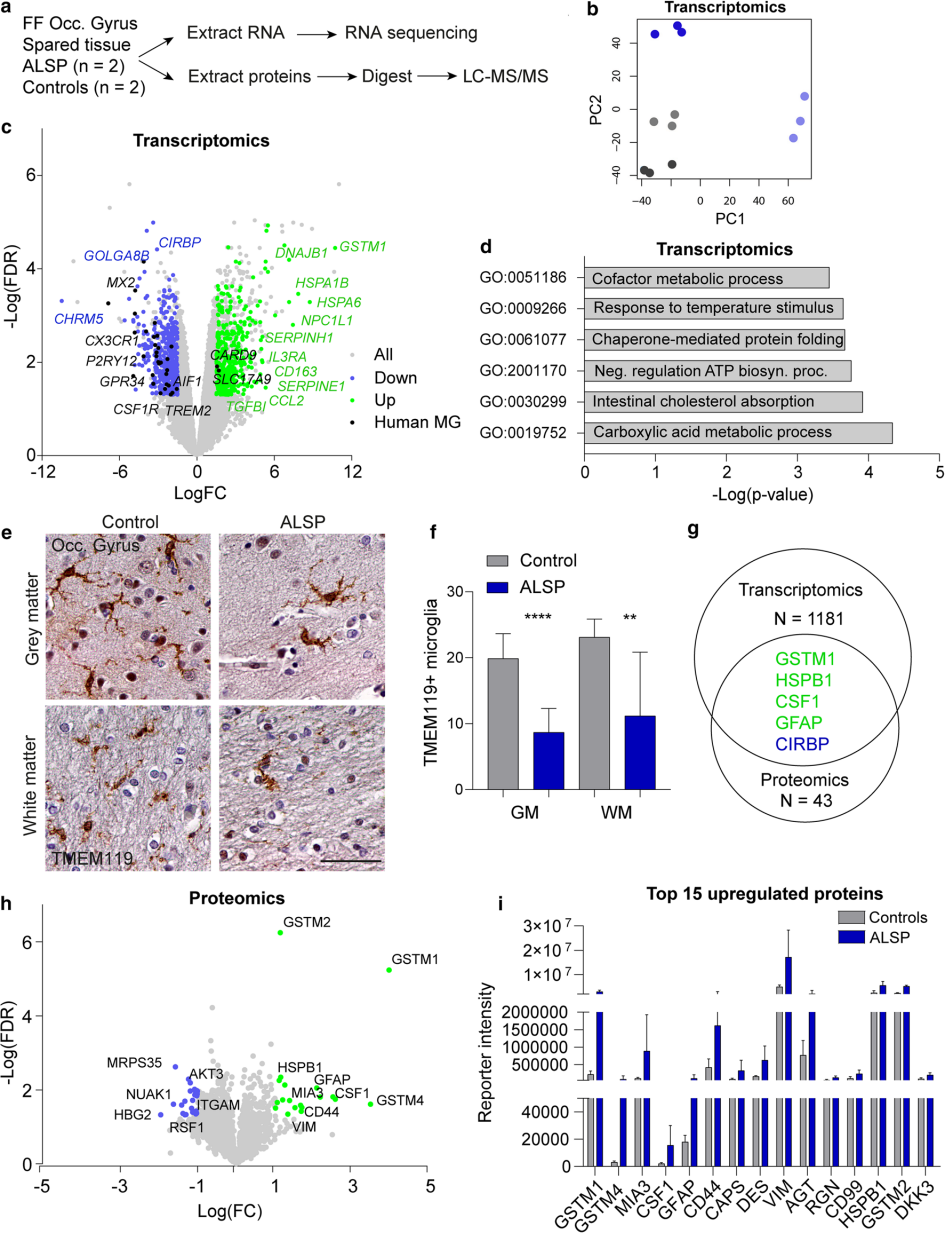
Transcriptomic and proteomic analysis of least-affected tissue of ALSP patients and controls. **a** Schematic representation of the experimental design. Fresh–frozen (FF) post-mortem brain tissue of the occipital gyrus was used to extract RNA and proteins. *N* = 2/group. **b** PCA plot of transcriptomics of the control (grey, *n* = 2) and ALSP tissue (blue, *n* = 2). **c** Volcano plot showing all genes picked up by bulk RNA sequencing (grey), and DEG (*n* = 1181) (downregulated: blue; upregulated: green). Black dots represent human microglia (MG) genes found differentially expressed in ALSP tissue [38]. DEG: FDR < 0.05, − 1.5 < LogFC > 1.5. **d** Representative graph of GO pathway analysis of the DEG. **e** Representative TMEM119 IHC images of the tissue (occipital gyrus) of controls (*n* = 2) and late-stage ALSP patients (*n* = 2) in grey matter (top row) and white matter (bottom row). **f** Quantification of TMEM119 + microglia in grey (left) and white matter (right) of occipital gyrus, count based on five images/individual. **g** Venn diagram showing the number of genes and proteins found to be differentially expressed in transcriptomics (*n* = 1181) and proteomics (*n* = 43), and the overlapping genes found in both data sets (*n* = 5). Green: upregulated, blue: downregulated. **h** Volcano plot showing all proteins detected by LC–MS/MS (grey) and differentially expressed proteins (*n* = 43) (downregulated: blue; upregulated: green). Differentially expressed proteins: FDR < 0.05, − 1 < LogFC > 1. **i** Representative graph of the normalized reporter intensity of the top 15 most upregulated proteins in ALSP (blue) and control tissue (grey). False-discovery rate (FDR), fold change (FC), grey matter (GM), occipital gyrus (Occ. gyrus), white matter (WM). Brown–Forsythe ANOVA with Dunnett’s multiple comparison test was performed to test for significance in the microglia count analysis (*p* < 0.05). **p < 0.01 ****p < 0.0001. Error bars represent SD. Scale bars equal 50 μm

Having shown in ALSP post-mortem brain tissue and in an in vivo model that CSF1R missense variants lead to microglial depletion, we aimed to explore molecular and cellular changes in ALSP that correlated with microglial loss. We previously showed that in two late-stage ALSP patients white matter of the occipital gyrus was relatively spared and had lower microglial density based on IHC of IBA1 and HLA-DR [83]. We reasoned that by analyzing this relatively spared tissue, we could identify more subtle changes correlating with an early stage of disease and microglial loss that could be independent from severe white matter pathology and/or neurodegeneration. We extracted total mRNA and protein from fresh–frozen post-mortem tissue of the occipital gyrus of two ALSP patients and two age- and sex-matched controls to perform bulk RNA sequencing and LC–MS/proteomics (Fig. 5a, Suppl. Fig. 4i, Online Resources). Patient and control RNA-seq data clustered independently (Fig. 5b) with patients showing reduced *CSF1R* expression (Fig. 5c). In total, 1181 genes were differentially expressed between ALSP and control brain tissue (logFC <1.5, FDR < 0.05) (Suppl. Table 3, Online Resources). ALSP brain tissue showed downregulation of genes highly expressed in microglia, including *P2YR12*, *CX3CR1*, *MX2*, *GPR34*, *AIF1* and *TREM2* (Fig. 5c), consistent with the reduced presence of (homeostatic) microglia [38]. Conversely, there was a minor upregulation of microglial genes related to MS and AD (e.g., *RUNX3*, *GPR146*, *CXCR4*; Suppl. Fig. 4a, b, Online Resources) [108, 122]. In addition, *CD163* was upregulated, consistent with our neuropathological examination of ALSP frontal gyrus tissue (Fig. 5c, Suppl. Fig. 1d, e, Online Resources). Reduced microglial gene expression is consistent with our previous findings, showing fewer IBA1 + cells by IHC staining in relatively spared occipital gyrus [83]. To test at the protein level whether a more microglia-specific myeloid marker would also show fewer marked cells, we stained for TMEM119 and observed reduced numbers of TMEM119 + microglia in ALSP grey and white matter (Fig. 5e, f). Altogether, these findings support the idea that depletion of (homeostatic) microglia is not limited to severely affected brain tissue.

In addition to microglial changes, several heatshock proteins/chaperone-encoding transcripts were upregulated, including *HSPA1B*, *DNAJB1*, *HSPA6* and *DNAJB4* as well as astrocytic genes *GFAP* and *GSTM1* (Fig. 5c). Surprisingly, only a few oligodendrocyte-related genes showed minor changes in expression (e.g., downregulated: *OPALIN*, *CARNS1*, *HMGCS1*, *KHNH8*, *MAG* and *MOBP*; upregulated: *VCAN*, *CA2* and *PDZRN4*) (Suppl. Fig. 4c, Online Resources) [64, 66]. Finally, we noticed differential expression of several extracellular matrix (ECM) genes, including ECM core proteins (e.g., upregulated: *VCAN*, *CHAD*, *PRELP* and *TGFBI*; downregulated: *SVEP1*, *COL24A1*, *COL9A3*, *COL15A1* and *GLDN*) and ECM modulators (e.g., upregulated: *SERPINE1*, *SERPINEH1*, *SERPINF2*, *ADAMTS9*, *ADAMTS2* and *PLOD3*; downregulated: *ADAMTS14*, *CD109* and *ADAM28*) [77] (Suppl. Fig. 4d, e). Gene ontology pathway analysis showed dysregulation of several metabolic processes, including lipids and chaperone-mediated protein folding, of which the latter is typical for an elevated stress response (Fig. 5d, Suppl. Table 4, Online Resources).

In our proteomic analysis, 43 proteins were found to be significantly different in ALSP brain tissue (LogFC <1, FDR < 0.05), of which five overlapped with transcriptomic data, both gene and direction of change: GSTM1, HSPB1, CSF1, GFAP and CIRBP (Fig. 5g, Suppl. Table 5, Online Resources). The increased abundance of M-CSF1, the ligand for CSF1R, could possibly be due to reduced receptor-mediated endocytosis as previously found in *Csf1r*-/- mice, leading to higher ligand levels (Fig. 5h) [29]. In addition, ALSP tissue showed a downregulation of microglial ITGAM/CD11b and GSTK1 (Fig. 5h, Suppl. Fig. 4f, Online Resources). Intriguingly, upregulated proteins included mainly proteins expressed by astrocytes (e.g., GFAP, CD44, VIM and HSPB1) (Fig. 5h, i, Suppl. Fig. 4 g, Online Resources) [35, 126]. To pick up more subtle changes, we selected proteins based on less stringent protein abundance criteria (LogFC <0.3, FDR < 0.05) and compared these to published gene sets. Some differential proteins were related to the ECM, including a minor increase in ECM core protein brevican (LogFC: 0.455; FDR: 0.016) (Suppl. Fig. 3 h, Online Resources) [77]. In summary, transcriptomics and proteomics show additional signs consistent with fewer microglia, an elevated stress response, altered metabolic pathways, and particularly the increased abundance of several astrocytic proteins in microglia-depleted brain areas.

### Abnormal astrocytic morphology and astrocyte-specific expression of GSTM1 in ALSP

**Fig. 6:**
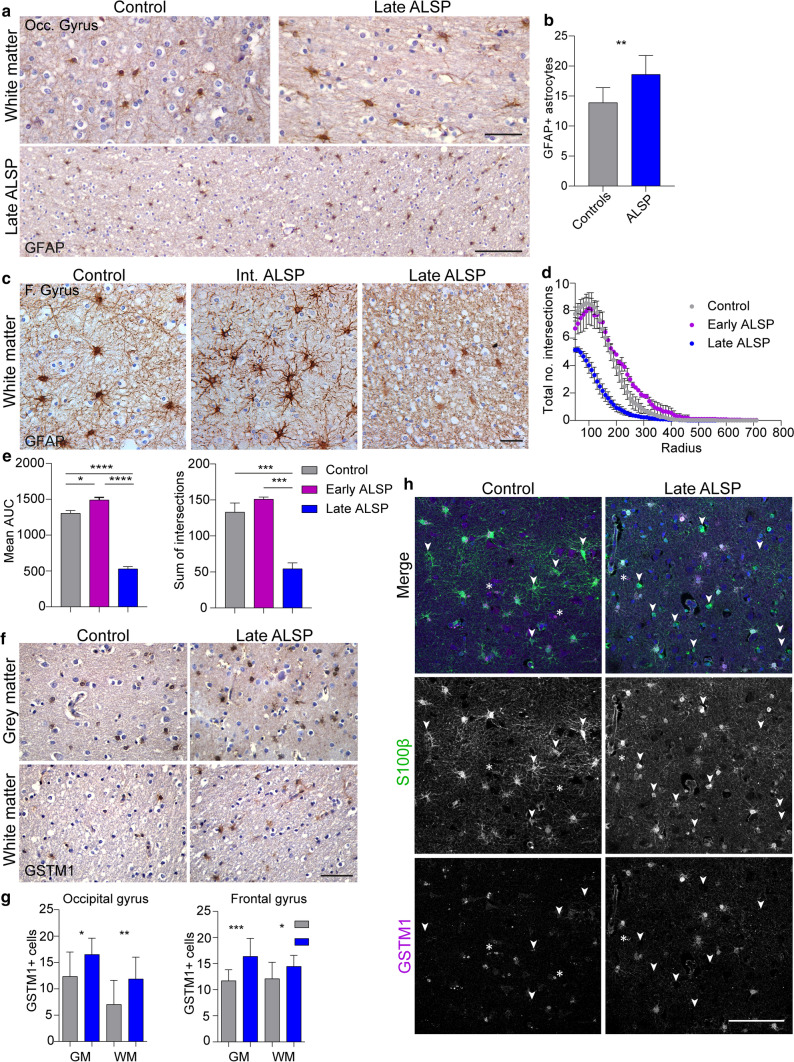
Abnormal astrocytic morphology and astrocyte-specific expression of GSTM1 in ALSP. **a** Representative images of occipital gyrus tissue of late-ALSP patients (*n* = 2) and controls (*n* = 2) stained with GFAP to visualize astrocytes in the white matter. **b** Quantification of the number of GFAP + astrocytes in the white matter of the occipital gyrus, based on five images/donor (*n* = 2/group). **c** Representative images of the white matter of the frontal gyrus stained with GFAP in intermediate-stage ALSP patients (*n* = 2), late-stage ALSP patients (*n* = 6) and controls (*n* = 3). **d** Sholl plot of GFAP + astrocytes found in the white matter of the frontal gyrus showing the total number of intersections per radius in intermediate and late ALSP vs control. **e** Quantifications of Sholl analysis on GFAP + astrocytes showing the mean area under the curve (AUC) (left) and total sum of intersections (right). **f** Representative images of GSTM1 staining of occipital gyrus tissue of late-ALSP patients (*n* = 2) and controls (*n* = 2) stained in the grey matter (top), white matter (bottom) and frontal gyrus of late-ALSP patients (bottom). **g** Quantification of the number of GSTM1 + cells in the grey and white matter of the occipital gyrus (left) and the frontal gyrus (right). Each dot represents the total number of GSTM1 + cells in one image, six images/donor (*n* = 2/group). **h** Representative images of frontal gyrus tissue of ALSP patients (*n* = 2) and controls (*n* = 2) stained with S100β (green), to visualize astrocytes, and GSTM1 (magenta). White arrows represent S100β + GSTM1- astrocytes; white asterisks represent S100β- GSTM1 + cells. Frontal (F.), grey matter (GM), Occipital (Occ.), white matter (WM). One-way or two-way ANOVA test was preformed to test for significance (*p* < 0.05). Error bars represent SD and SEM (**d**). *p < 0.05, **p < 0.01 ***p < 0.001 ****p < 0.0001. Scale bars equal 10 μm (**a**, upper panel), 100 μm (**a**, lower panel; **h**), 20 μm (**c**, **f**)

Astrogliosis has been reported in ALSP case studies [11, 58], and is considered a general neuropathological hallmark, often characterized by IHC for the highly abundant astrocytic protein GFAP [35]. Since proteomics revealed a profound upregulation of astrocyte-related proteins, we performed IHC to confirm these findings in situ. In white matter of the relatively spared occipital gyrus, we observed more GFAP + astrocytes (Fig. 6a, b) and a more hypertrophic morphology of S100β + astrocytes in white matter in ALSP compared to controls (Fig. 8a, Suppl. Fig. 5c, d, f). Sholl analysis for morphology of astrocytes in severely affected frontal gyrus revealed that GFAP + astrocytes in late-ALSP white matter had fewer cellular processes and increased hypertrophy (Fig. 6c–e), which we confirmed in grey and white matter using the pan-astrocytic marker ALDH1L1 [15, 79] (Suppl. Fig. 5a, b), and slightly more, longer GFAP + astrocytic processes in intermediate-stage patient tissue (Fig. 6c–e). In addition, GSTM1, one of the most abundant astrocytic proteins in mice, was highly upregulated both in RNA-seq and proteomics data [103]. In mice, GSTM1 promotes astrocyte-mediated microglial activation during brain inflammation [50]. ALSP brain tissue showed more GSTM1 + cells in grey and white matter of both the occipital gyrus and the more affected frontal gyrus (Fig. 6f, g). GSTM1 appeared to be highly expressed in astrocytic cell bodies and fine cellular processes, which was barely observed in controls (Fig. 6f, g). Co-staining of GSTM1 with S100β, which is highly expressed in mature astrocytes in grey and white matter [96], confirmed that GSTM1 was highly expressed by astrocytes (Fig. 6h). Nevertheless, several S100β + cells were GSTM1-, indicating that GSTM1 was only expressed in a subset of astrocytes (Fig. 6h, indicated by white arrows). To test whether elevated GSTM1 was specific to ALSP, we examined GSTM1 expression in post-mortem frontal gyrus tissue of AD and frontotemporal dementia (FTD) patients. Both showed abundant presence of GSTM1 + cells with an astrocyte-like morphology in grey and white matter (Suppl. Figure 6, Online Resources), indicating that high GSTM1 expression could be shared among neurodegenerative diseases. Altogether, both relatively spared and severely affected tissue of late-stage ALSP patients show an altered astrocytic phenotype.

### Altered astrocytic phenotype in zebrafish missense mutants indicates compensatory astrocytic endocytosis in early development

**Fig. 7:**
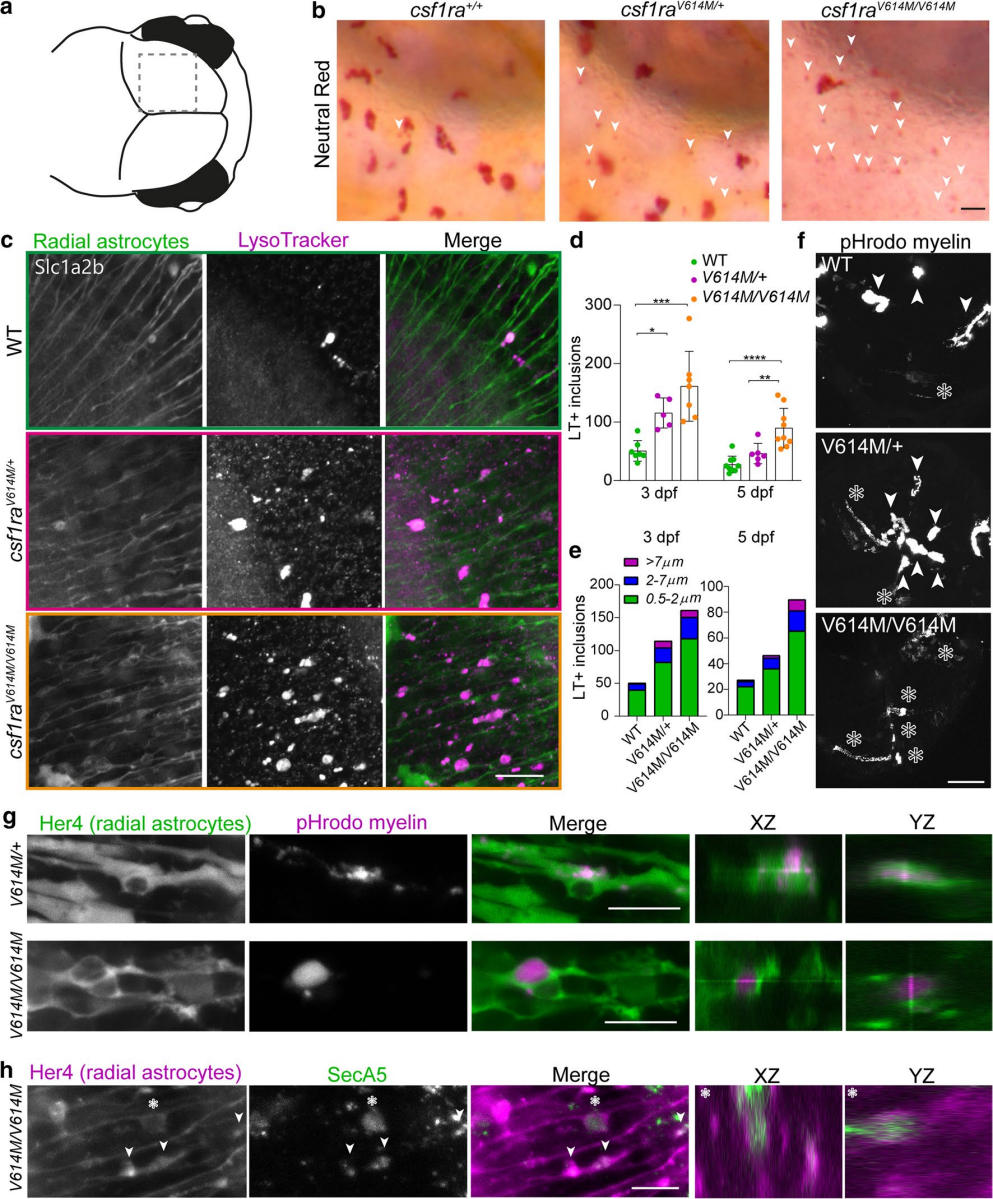
Astrocytic phenotype in heterozygous and homozygous missense zebrafish mutants indicates compensatory astrocytic endocytosis in early development. **a** Schematic image of the midbrain region imaged here. **b** Representative images of NR staining of half of the midbrain of *csf1ra*^+/+^, *csf1ra*^V614M*/*+^ and *csf1ra*^V614M*/*V614M^ at 3 dpf. White arrows show small NR + dots in the region where astrocytes reside. **c** Representative images of LysoTracker staining (magenta) of *csf1r* WT, *csf1ra*^V614M*/*+^ and *csf1ra*^V614M*/*V614M^ in a *slc1a2b*:Citrine background (green), visualizing radial astrocytes, in vivo at 3 dpf*.* **d** Quantifications of the number of LT + inclusions within radial astrocytes in the midbrain at 3 dpf in *csf1r*^WT^ (*n* = 7, 9), *csf1ra*^V614M*/*+^ (*n* = 5, 6) and *csf1ra*^V614M*/*V614M^ (*n* = 7, 9). **e** Quantifications of the number of LT + inclusions within radial astrocytes in the midbrain at 3 dpf (left) and 5 dpf (right), per category based on diameter of the inclusion (0.5–2 μm: green; 2–7 μm: blue; < 7 μm: purple). **f** Representative images of a maximum projection of a time-lapse video (3 h, 18–21 hpi), showing engulfment of pHrodo-labeled myelin by microglia (white arrows) and by radial astrocytes (white asterisks). **g** Representative images of pHrodo-labeled myelin inclusions (magenta) within radial astrocytes (green) in the midbrain of *csf1ra*^V614M*/*+^ and *csf1ra*^V614M*/*V614M^ larvae at 4 dpf, 18 h post-injection (hpi). **h** Representative images of apoptotic cell particle (SecA5-mVenus + , green) inclusions within radial astrocytes (magenta) in the midbrain of *csf1ra*^V614M*/*V614M^ larvae at 3 dpf. One-way ANOVA or two-way ANOVA test was preformed to test for significance (*p* < 0.05). Error bars represent SD. *p < 0.05, **p < 0.01 ***p < 0.001. Scale bars equal 15 μm (**b**, **c**), 50 μm (**f**), 10 μm (**g**, **h**)

Since our unbiased screen and IHC examination revealed that altered astrocytes may correlate with loss of microglia, we wondered whether astrocytes could play a role in the early pathogenesis of ALSP. In zebrafish, radial glia are functionally homologous to mammalian astrocytes and are referred to as radial astrocytes [18, 76]. Therefore, we investigated radial astrocytes in early brain development of *csf1r*^ALSP^ larvae. In the *csf1ra*^*V614M/*+^ and *csf1ra*^*V614M/V614M*^ zebrafish mutants, we observed small NR + inclusions in the midbrain where radial astrocytes reside, which we hardly detected in controls (Fig. 7a, b). We observed similar small NR + dots in the midbrain of the progeny of *csf1ra*^ex17∆5/+^ with *csf1ra*^A784V/+^ mutants, but only in *csf1ra*^A784V/+^ and *csf1ra*^ex17∆5/A784V^ larvae where we showed fewer microglia (Suppl. Fig. 7a, Online Resources, Fig. 3b, c). Based on their small size, we presumed that these NR + inclusions were not inside microglia, but instead, were endocytosed by radial astrocytes under microglia-depleted conditions, reminiscent of the astrocytic compensatory phagocytosis reported in microglia-depleted mice [30, 57]. We, therefore, used transgenic *slc1a2b*:Citrine zebrafish, which labels radial astrocytes expressing the main glutamate transporter Eaat1, known as GLT1 in mice, and LysoTracker Red (LT) to visualize lysosomes [61]. This revealed more LT + inclusions in radial astrocytes in *csf1ra*^*V614M/*+^ and *csf1ra*^*V614M/V614M*^ larvae from 3 dpf than in controls, and more inclusions with a large diameter (> 2 µm) (Fig. 7b, c, e). Similarly, we found more LT + inclusions in radial astrocytes in *csf1ra*^A784V/+^ and *csf1ra*^ex17∆5/A784V^ larvae, but not in *csf1ra*^ex17∆5/+^, at 3 dpf (Suppl. Fig. 7b, c, Online Resources). Differences in LT-staining were only observed in the midbrain, where most microglia typically reside at this time point (Suppl. Fig. 8a, Online Resources). In addition, large inclusions were exclusively found in *slc1a2b*:Citrine + radial astrocytes and not in neurons expressing Elavl3 (Suppl. Fig. 8b, Online Resources) [3]. At 5 dpf, the lysosomal inclusions in *csf1ra*^*V614M/*+^ larvae did no longer differ to controls, but did differ from *csf1ra*^*V614M/V614M*^ (Fig. 7d, e).

As myelin debris accumulated in white matter lesions is usually degraded by microglia through phagocytosis, we hypothesized that astrocytes may at least in part compensate for that role. Hence, we proceeded with intracerebral injections of pHrodo-labeled human myelin in *csf1ra*^*V614M/*+^, *csf1ra*^*V614M/V643M*^ and control larvae with a *her4*:eGFP transgenic background visualizing radial astrocytes, to study whether these cells would show enhanced endocytosis of myelin under a microglia-depleted condition [44, 124]. We used pHrodo-labeled myelin to study the uptake of myelin in acidic particles, including lysosomes. Indeed, radial astrocytes in *csf1ra*^*V614M/*+^ and *csf1ra*^*V614M/V614M*^ larvae showed uptake of pHrodo-labeled myelin, while in controls, we only observed accumulation of myelin in big phagosomes, likely within microglia (Suppl. Movie 1, Fig. 7f, g). Interestingly, heterozygous missense mutants showed a combination of microglial and radial astrocytic uptake of pHrodo myelin while homozygous missense mutant exhibited no sign of microglial uptake (Suppl. Movie 1, Fig. 7f). To further investigate astrocytic endocytosis under microglia-depleted conditions, we obtained in vivo images of *csf1ra*^*V614M/V614M*^ larvae in a background of transgenic marker *her4.1*:mCherry, expressed in radial astrocytes, co-expressing an Annexin 5-based transgenic fluorescent marker of apoptotic particles (ubi:secA5-mVenus) [60, 61]. We observed SecA5-mVenus + apoptotic cell particle inclusions within radial astrocytes in the *csf1ra*^*V614M/V614M*^ but not in control larvae, indicating the engulfment of apoptotic fragments by radial astrocytes (Fig. 7h). Thus, ALSP-causing missense mutant zebrafish larvae showed an elevated endocytic astrocytic phenotype at 3 dpf, which could be a compensatory adaptation to decreased microglial endocytosis. However, at 5 dpf, the number of lysosomal inclusions in heterozygous missense mutants was comparable to controls.

### Elevated lysosomal vesicles in astrocytes and engulfment of myelin debris indicate compensatory astrocytic endocytosis with loss of microglia in ALSP patients

**Fig. 8:**
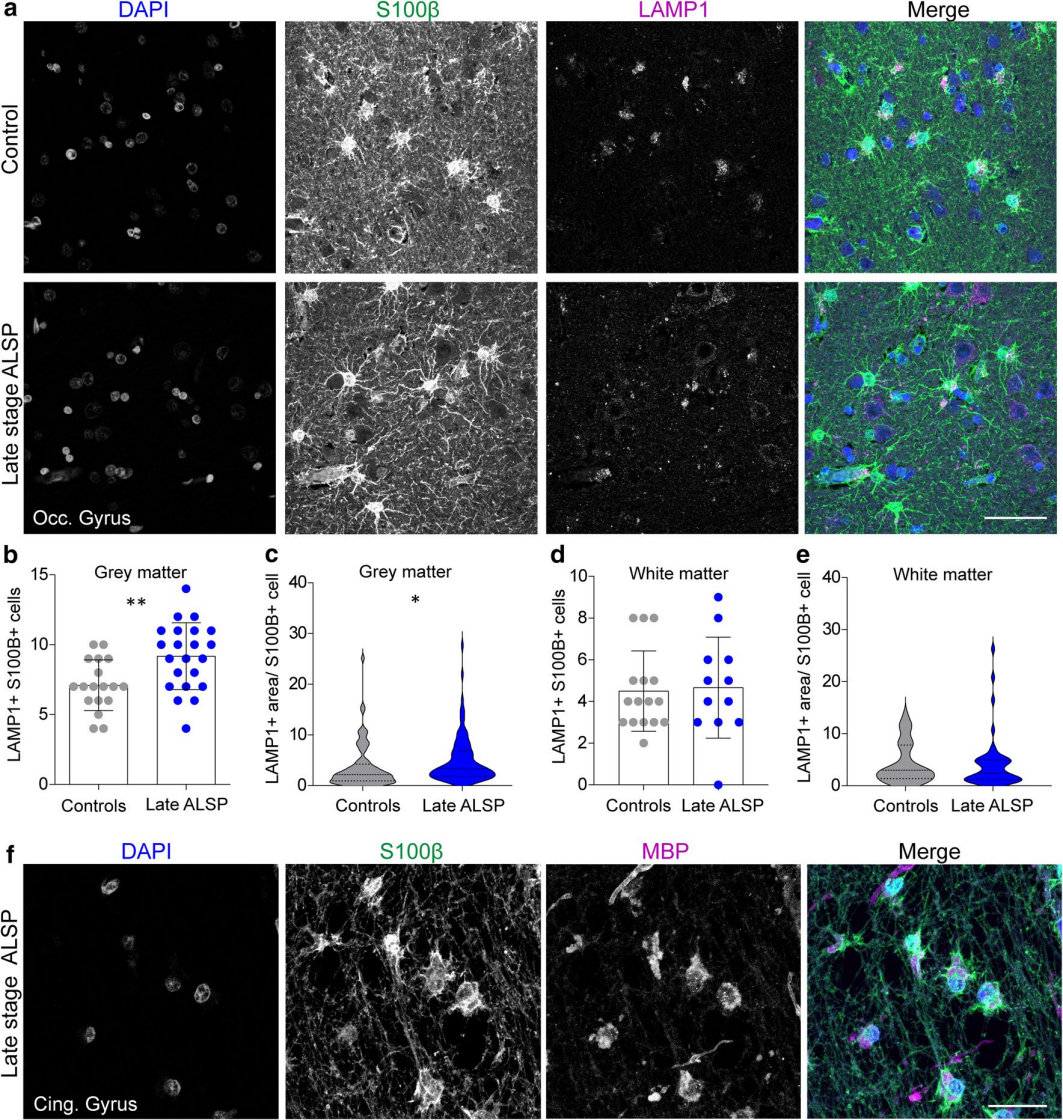
Elevated lysosomal vesicles in astrocytes and engulfment of myelin debris indicate compensatory astrocytic endocytosis in ALSP. **a** Representative confocal images of grey matter of the occipital gyrus tissue of late-stage ALSP patients and controls stained with DAPI (nuclei, blue), S100β (astrocytes, green) and LAMP1 (lysosomes, magenta). **b, c, d, e** Quantification of the number of LAMP1 + S100β + astrocytes in grey matter (**b**) and white matter (**d**), and the LAMP1 + area per S100β + astrocyte in grey matter (**c**) and white matter (**e**) of the occipital gyrus of late-stage ALSP patients (*n* = 2) and controls (*n* = 2). **f** Representative confocal images showing uptake of MBP + myelin (magenta) by S100β + astrocytes (green) in a white matter lesion in the cingulate gyrus of late stage ALSP patients. Student *t* test was preformed to test for significance (*p* < 0.05). Error bars represent SD. *p < 0.05, **p < 0.01 ***p < 0.001. Scale bars equal 50 μm (**a**) and 30 μm (**f**)

To investigate whether an increased astrocytic endocytic phenotype would also be present in ALSP, we stained occipital gyrus tissue from ALSP patients and controls with S100β and LAMP1, visualizing, respectively, mature astrocytes in grey and white matter and lysosomal vesicles [6, 96]. In grey matter of ALSP tissue, there was an increased number of S100β + /LAMP1 + cells (Fig. 8a, b). In addition, the total area of LAMP1 per S100β + cell was increased in the grey matter of ALSP tissue (Fig. 8c), which was not observed in white matter (Fig. 8d, e). In white matter lesions of the cingulate gyrus, we observed many S100β + astrocytes that showed high cytoplasmic staining of myelin basic protein (MBP), a highly abundant myelin protein (Fig. 8f). Altogether, findings in ALSP brain tissue were consistent with enhanced astrocytic lysosomal activity and myelin uptake by astrocytes, possibly reflecting a compensatory endocytic response by astrocytes in a microglia-depleted state.

## Discussion

Here, we establish by independent approaches that microglial depletion due to dominant-acting CSF1R missense variants is an early hallmark of ALSP. Furthermore, by multi-omic analyses in post-mortem ALSP brain tissue and in vivo experimentation in zebrafish mutants, we explored putative consequences of microglial depletion, identifying an altered astrocytic phenotype characterized by increased endocytosis. We also demonstrate that in zebrafish heterozygous pathogenic CSF1R missense variants, but not a heterozygous null allele, result in microglial depletion already in embryonic brain development. This provides in vivo mechanistic evidence that such variants act dominantly, resulting in microglial depletion that may far precede onset of symptoms.

Previously, we found that there is a lower density of IBA1 + microglia in ALSP cortical tissue [83]. Our current analyses of brain tissue of late- and intermediate-stage ALSP extend these findings and show overall reduced homeostatic P2RY12 + and TMEM119 + microglia, particularly in white matter. Consistently, transcriptomic analyses of relatively spared occipital gyrus revealed downregulation of microglia-specific genes. Furthermore, our findings from two ALSP-causing CSF1R missense variants in genome-edited zebrafish models showed microglial depletion in early development. These findings provide in vivo evidence that microglial depletion may far precede onset of symptoms. Only in intermediate-stage patients, numbers of TMEM119 + microglia did not differ from controls in grey matter, in contrast to P2RY12 + microglia. A possible explanation for this is that TMEM119 is also expressed on amoeboid immunoreactive microglia, whereas P2RY12 is considered more exclusive to homeostatic ramified microglia [99, 127]. Therefore, subsets of microglia could be affected differently or possibly P2RY12 would be more sensitive to changes in microglial homeostasis. Previously, we and others observed a clustered distribution of microglia in multiple brain regions in ALSP, where some areas were completely devoid of IBA1 + microglia [83,^113^]. Kempthorne et al. (2020) also identified reduced expression of microglial genes in ALSP, but speculated that this was due to a loss of homeostatic gene expression rather than a loss of microglia [52]. However, this does not explain the local density differences we observe, including local clustering. A shortage of microglia in one area could perhaps lead to altered distribution by attracting microglia away from other areas, driven by an imbalance in the need and availability of microglia. Clustering of microglia, e.g., in response to cell death or plaques, is typical for various neuropathologies. However, in ALSP, these clusters of immunoreactive microglia, which could develop later in disease as a response to pathology, may further drive depletion in areas with an already low density of microglia by pulling away microglia towards areas of pathology. Microglial depletion caused by recruitment to areas in need could be further reinforced by the defective microglial proliferation. In *csf1r*-deficient zebrafish, we also observed this phenomenon, where microglial recruitment after neuronal ablation involved migration of resident microglia to the site of injury, in the absence of microglial proliferation [83]. Our data indicate that ALSP involves both a loss of homeostatic microglia as well as a general microglial depletion, predominantly in the white matter. Of note, we observed clusters of CD163 + macrophages/microglia, predominantly in white matter, and an upregulation of *CD163* in ALSP tissue. CD163 is associated with an anti-inflammatory microglial signature driving remyelination and found in active MS lesions [72,125]. Since we also noticed brain areas with few CD163 + cells, the CD163 + clusters may again be a consequence of an overall depletion of microglia. Notably, we cannot exclude the possibility that a proportion of these CD163 + cells are infiltrating peripheral macrophages. Altogether, our findings further support the concept of HSCT as a treatment for ALSP, which may act by repopulation of a depleted microglial niche, comparable to the effect of HSCT in adrenoleukodystrophy where pre-lesion areas are also characterized by reduced numbers of microglia [12].

We demonstrate that 2 independent pathogenic CSF1R missense variants in the zebrafish *csf1ra* locus act dominantly in decreasing density of microglia, whereas a null allele, leading to protein production from only one allele, had no effect. This dominant effect in vivo is further supported by recent observations in mice carrying a heterozygous ALSP-causing CSF1R missense variant (*Csf1r*^E631K/+^), showing reduced numbers of microglia [109]. Also, in vitro biochemical experiments show that pathogenic CSF1R missense variants act dominantly on CSF1R signaling, and parents of bi-allelic patients with heterozygous haploinsufficient *CSF1R* variants have not been noted to develop brain disease [40, 50, 55, 74, 81, 92, 114]. Furthermore, 86% of pathogenic variants in *CSF1R* are missense variants, predominantly located in the TKDs, whereas only 4% of pathogenic variants in *CSF1R* are frameshift variants (Suppl. Table 2, Online Resources). As most of these frameshift variants are located in the C-terminal end, they could in fact also lead to a dominantly acting truncating protein. It is not clear whether any ALSP case can be explained by haploinsufficiency. Indeed, haploinsufficient *Csf1r*+/- mice show no decrease in microglia, whereas complete knockout mice have no microglia [7, 19, 34]. Therefore, although such models can be very useful, they should be treated with caution when used to study ALSP. What is intriguing, however, is our observation of the differential effects on the number of microglia between CSF1R missense variants. As patients with the same *CSF1R* variant show major differences in age of onset and disease severity, it is likely that environmental factors and/or genetic factors play a disease-modifying role. This obscures possible genotype/phenotype relationships, and therefore, it has remained unclear whether pathogenic *CSF1R* variants correlate with ALSP age of onset and severity. Our results suggest that there likely are variant-specific effects contributing to the level of microglial depletion, and by extension possibly also disease severity. Together with possible environmental or genetic modifying factors, this could explain the variance in age of onset and disease course of ALSP patients.

Through unbiased analyses, we identified an altered astrocytic phenotype in ALSP brain tissue and in zebrafish already during embryonic development. In ALSP tissue of severely affected frontal gyrus, but also in relatively spared white matter of the occipital gyrus, astrocytes were more hypertrophic, which is associated with elevated reactivity [35]. Thus, astrocytic abnormalities may be an early consequence of microglial loss—although we cannot exclude that they originate from another cause—and possibly also play a role in early ALSP pathogenesis. Of note, since zebrafish astrocytes morphologically differ from those in mammals, it is not yet evident to what extent the phenotype we observed in radial astrocytes is analogous to that of astrocytes in ALSP, but it nevertheless gives an indication of early embryonic changes correlating with microglial depletion in the vertebrate brain. Notably, radial astrocytic lysosomal inclusions in heterozygous missense zebrafish mutants were transiently present, which could imply that the astrocytic compensatory response is needed temporarily when the phagocytic demand is high, since there is an increased number of apoptotic cells in the zebrafish brain around 3 dpf [45]. The astrocytic phenotype in our zebrafish mutants is reminiscent of the compensatory endocytic response by astrocytes in microglia-depleted mice [30, 57]. Indeed, *csf1ra*^*V614M/*+^ larvae showed increased endocytosis of myriad substances, including NR dye, apoptotic particles and even myelin. We also observed phagocytized myelin debris inside astrocytes in ALSP brain tissue. This is reminiscent of observations in demyelinating lesions in MS and metachromatic leukodystrophy [90], although the amount of myelin debris within astrocytes appeared remarkably high in ALSP patient tissue. Astrocytes needing to process and degrade phagocytic waste could lead to neglected homeostatic functions, including energy supply to neurons and oligodendrocytes, blood–brain-barrier integrity, and uptake of neurotransmitters [16, 42, 54, 68, 84, 118]. Several leukodystrophies, e.g., Alexander’s disease, Megalencephalic leukoencephalopathy with subcortical cysts and Vanishing White Matter disease, are caused by genetic variants in astrocyte-associated genes or involve astrocytic dysfunction as a pivotal pathogenic mechanism [65, 121]. Therefore, abnormal astrocytic phenotypes appear sufficient to cause leukodystrophy. On the other hand, astrocytic endocytosis may partially rescue the shortage of microglial debris clearance, which could be important for prolonging the maintenance of a healthy CNS. Hence, compensatory endocytosis by astrocytes could have both beneficial and detrimental effects on brain health, but future research is needed to further establish this.

The importance of understanding the effects of microglial depletion in the human brain is increasingly relevant as multiple clinical trials are focusing on CSF1R inhibitors to deplete microglia in brain disease. We focused our omics analysis on occipital gyrus tissue, where white matter is relatively spared and pathology is relatively mild, allowing us to detect moderate changes that correlate well with an early stage of disease. Tissue analyzed included both grey and white matter, to allow detection of possible changes in both grey and/or in white matter. Of note, since ALSP is a rare disease and high-quality post-mortem brain tissue is scarce, our multi-omics analyses were performed on brain tissue of two ALSP patients who underwent autopsy with a very short post-mortem delay. It is unclear whether our analyses on two patients fully represent the general ALSP population. Nevertheless, we could replicate findings from these datasets by IHC in multiple patients and in zebrafish models. We found relatively few changes by transcriptome and proteome analysis in ALSP brain tissue, including downregulation of microglial genes, an elevated stress/heat shock response and increased abundance of astrocytic proteins. In particular, glutathione S-transferases (GSTs), including GSTM1, were upregulated at the transcript and protein level. In mice, GSTM1 is secreted by astrocytes to stimulate a pro-inflammatory microglial response [51, 103]. As we find abundant GSTM1 expression in astrocyte-like cells in AD and FTD post-mortem brain tissue, astrocytic GSTM1 upregulation may be present across neurodegenerative diseases. We also found differential expression of ECM proteins in ALSP brain. Recent studies reported microglial ECM remodeling, including pruning of aggrecan and brevican, of which the latter is slightly increased in ALSP brain tissue [7, 23, 80, 112]. Notably, we cannot exclude whether these changes might in part reflect region-dependent effects rather than disease stage differences, and in fact others have observed regional microglial changes in ALSP [52]. In our proteome and transcriptome analysis, we cannot distinguish cortical or white matter-specific changes. Nevertheless, based on immunohistochemical staining on the same, relatively spared tissue, we did not detect differences that were exclusive to either cortex or white matter, except for increased expression of LAMP1 + in S100β + astrocytes only in cortical tissue, a more hypertrophic morphology of S100β + astrocytes in spared white matter and astrocytic engulfment of myelin in demyelinating lesions. This could indicate that astrocytes in white versus grey matter respond differently to the lack of microglia. Microglial depletion could influence white matter health by multiple possible independent effects, including possibly (indirectly) affecting astrocytic functioning through their compensatory endocytic response to elevated phagocytic demand. Our results here provide initial steps to further understand consequences of microglial depletion in the human brain.

In addition to having fewer microglia and altered radial astrocytes, heterozygous *csf1ra*^*V614M/*+^ zebrafish also had fewer sox10:RFP + oligodendrocytes in the hindbrain, but normal myelination and normal locomotor activity, whereas homozygous mutants showed a smaller myelinated area and reduced locomotor behavior. *Csf1r*-/- mice also present with reduced oligodendrocyte cell numbers, although the overall myelin patterns in both *Csf1r*^E631K/+^ mice and *Csf1r*-deficient mice and rats appear relatively normal [34, 91, 109].  Cunha et al. (2020) reported normal myelin content in the zebrafish *csf1r*-mutant spinal cord, as we observed in larvae, but an impaired remyelination capacity [26]. Thus, as mentioned above, there could be regional- and density-dependent effects of microglial depletion on myelin health in zebrafish. Overall, it is currently unknown to what extent the clinical manifestation of ALSP is preceded by cellular phenotypes as we observe in zebrafish.

Zebrafish as a neurobiology model allows exploring cells in embryonic development, in their natural environment by non-invasive in vivo examination. This has led to new fundamental insight in glial biology [11, 71, 78]. In addition, options for genetic targeting have improved, including editing of specific genetic variants identified in human disease as we show here. Possible limitations, when used as a model for human brain disease, include dissimilarities at a more macro-scale such as the lack of a layered cortex and a low ratio of myelinated versus non-myelinated brain tissue, which may obscure pathology in adult animals. Furthermore, similar to rodent models, phenotypes in zebrafish models for leukodystrophies are relatively mild. For example, unlike in human disease, fully CSF1R-deficient mutant zebrafish and rats are viable, and have a mild phenotype [62, 91]. Therefore, comparisons between multiple models are needed to make conceivable conclusions on disease mechanisms. Nevertheless, zebrafish in particular are suitable to detect phenotypes that go unnoticed in other models, as we show here, to subsequently explore further in other model systems and post-mortem brain tissue.

Based on phenotypes described for complete loss of function of CSF1R in humans, rats, mice and zebrafish, most peripheral macrophages are also depleted, leading to various developmental abnormalities outside of the CNS [11, 29, 34, 50, 62, 81, 91]. As yet, it is still unclear what the effects of pathogenic *CSF1R* variants are on peripheral macrophages and CNS-myeloid cells other than microglia in ALSP patients, although two studies reported on effects on peripheral monocytes/macrophages, including impaired phagocytosis and reduced presence of SLAN + macrophages [41, 47]. Skeletal abnormalities are obvious in patients with bi-allelic CSF1R variants, but no skeletal abnormalities have yet been noted in ALSP patients [58]. Nevertheless, it is intriguing to investigate extra-cerebral tissues and macrophages in ALSP, including possible involvement of skull bone marrow-derived myeloid cells [25].

Concluding, we provide proof that depletion of (homeostatic) microglia in ALSP patients occurs as a result of pathogenic missense variants in CSF1R and additional evidence of the importance of microglia in the development and maintenance of a healthy brain. Furthermore, our findings indirectly support the beneficial effect of HSCT in ALSP—which may act by repopulation of an under-occupied microglial niche. It remains an open question how loss of microglia can result in severe white matter defects. We speculate that microglial depletion and/or dysfunction results in a cascade of effects on other brain cells, including astrocytes, due to lack of total phagocytic capacity and the disruption of microglial modulation of the CNS environment, that by an as yet unknown mechanism eventually leads to white matter abnormalities [11]. A better understanding of this cascade would improve our knowledge of ALSP and potentially other disorders where loss of microglial function is implicated. Taken together, further investigating ALSP and the effect of CSF1R missense variants in vivo may provide opportunities for improving microglia-focused treatment strategies and insight into the consequences of depletion of microglia, and possibly other macrophages, for the human brain.

## Supplementary Information

Below is the link to the electronic supplementary material.

Supplementary file1 Neuropathological examination of POLD patients and extra characterizations of microglia a Representative images of LFB and PAS staining of the frontal gyrus of POLD diagnosed patients (n=4), showing myelin loss and pigmented glia in the white matter b, c Representative NF (b) and HE (c) IHC images of the frontal gyrus of POLD diagnosed patients (n=4), showing axonal spheroids and pigmented glia in the white matter d Representative CD163 IHC images of the frontal gyrus of controls (n=3), intermediate-stage ALSP patients (n=2) and late-stage ALSP patients (n=6) in grey (top row) and white matter (bottom row) e Representative CD163 IHC images of the frontal gyrus of intermediate-stage ALSP patients showing clustered distribution of CD163+ cells in the white matter. Scale bars equal 50 μm (a, b, d), 100 μm (c) and 500 μm (e).Supplementary Fig. 2 Heterozygous ALSP-causing CSF1R missense variants cause defective proliferation of microglia a Representative image of mpeg1:GFP+ yolk sac macrophages (YSM) at 24 hpf. Dashed lines show outlines of the yolk sac (YS), the embryonic brain (EB) and the eye (E) b Quantification of mpeg1:GFP+ YSM on the yolk (left) and in the embryonic brain (right) at 24 hpf in csf1ra+/+ (n = 12) and csf1raV614M/+ (n=14) larvae c Maximum projection of a timelapse (32-47 hpf, 15 min interval) showing mpeg1:GFP+ YSM migration from the yolk sac into the embryonic brain d Quantification of mpeg1:GFP+ cells on the yolk sac (left) and in the embryonic brain (right) over time (32-47 hpf) in csf1ra+/+, csf1raA784V/+ and csf1raV614M/+ (n=4/group) e Representative images of single mpeg1:GFP+ microglia proliferating in vivo in the midbrain of zebrafish larvae, in a timelapse between 54-72 hpf f Quantification of proliferating microglia in the midbrain of csf1ra+/+ and csf1raV614M/+ larvae between 54 and 72 hpf (n=3/group). Eye (E), embryonic brain (EB), yolk sac (YS). One way or two way ANOVA, and Student t-test was preformed to test for significance (p<0.05). Error bars represent SD. * p<0.05. Scale bars equal 100 μm (a, c) and 10 μm (e).Supplementary Fig. 3 The number of sox10:RFP+ oligodendrocytes in different CNS regions at different timepoints a Representative images of the midbrain (top) and the hindbrain (bottom) of csf1ra+/+ (green), csf1raV614M/+ (purple) and csf1raV614M/V614M (orange) larvae in a sox10:RFP background, visualizing oligodendrocytes, at 3 dpf. Schematic of zebrafish embryonic midbrain (mb) and hindbrain (hb) b Quantification of the number of sox10:RFP + oligodendrocytes in the midbrain and hindbrain at 3 dpf in csf1ra+/+ (green), csf1raV614M/+ (purple) and csf1raV614M/V614M (orange) larvae c Representative images of the spinal cord of csf1ra+/+ (green), csf1raV614M/+ (purple) and csf1raV614M/V614M (orange) larvae in a sox10:RFP background, visualizing oligodendrocytes, at 3 dpf and 5 dpf d Schematic image of the position of the spinal cord in a zebrafish embryo e Quantification of the number of sox10:RFP+ oligodendrocytes in the spinal cord at 3 (left) and 5 dpf (right). Each dot represents one zebrafish, n=6/8. One way ANOVA was preformed to test for significance (p<0.05). * p<0.05 *** p<0.001. Error bars represent SD. Scale bars equal 100 μm.Supplementary Fig. 4 Comparing differentially expressed genes and proteins from transcriptomic and proteomic analysis to published RNA seq datasets a, b, c Genes related to: microglia in MS (a) [122], myeloid cells in AD (b) [108] and oligodendrocyte and oligodendrocyte precursor cells (c) [64, 66] in the DEG list of transcriptomic analysis d Volcano plot showing extracellular matrix (ECM) related genes found in the DEG in ALSP tissue (downregulated: blue; upregulated: green) [77]. Black dots represent ECM core proteins e Representative graph showing expression(count per million, CPM) of ECM regulators found among DEG in ALSP (blue) and control tissue (grey) [77] f, g, h Human microglia-related genes (f) [38], human astrocyte-related genes (g) [126] and ECM-related genes (h) [77] in the differentially expressed protein list of proteomic analysis i Clinical characteristics of donors of fresh-frozen brain tissue, used for RNA sequencing and mass spectrometry. Supplementary Fig. 5 Altered morphology of ALDH1L1+ and S100β+ astrocytes in late stage ALSP tissue a Representative ALDH1L1 IHC images of the frontal gyrus of control and late stage ALSP donors in the grey matter (top) and white matter (bottom) b Sholl analysis plot of ALDH1L1+ astrocytes in grey matter (top) and white matter (bottom) of the frontal gyrus showing the total number of intersections per radius in late ALSP vs control c Representative S100β IHC images of the occipital gyrus white matter of control and late stage ALSP donors (for grey matter, see Fig. 8a) d Sholl analysis plot of S100β+ astrocytes in grey matter (top) and white matter (bottom) of the occipital gyrus e, f Quantifications of Sholl analysis on ALDH1L1+ astrocytes in the frontal gyrus (e) and S100β+ astrocytes in the occipital gyrus (f) based on the mean AUC in grey matter (GM) and white matter (WM). One way or two way ANOVA test was preformed to test for significance (p < 0.05). Error bars represent SD. ** p < 0.01 **** p < 0.0001. Scale bars equals 50 μm.Supplementary Fig. 6 Abundant presence of GSMT1+ astrocyte-like cells in AD and FTD a, b, c Representative GSTM1 IHC images of post-mortem frontal gyrus tissue of AD (n = 2), FTD (n = 2) donors (a, c) and in control (n = 1) donor (b) in grey matter and white matter d Clinical characteristics of post mortem tissue of donors, all frontal gyrus tissue. Scale bars equal 100 μm (a), 20 μm (b), 10 μm (c).Supplementary Fig. 7 Astrocyte phenotype associated with enhanced endocytosis only present in heterozygous missense mutants and bi-allelic zebrafish mutants a Representative images of NR staining of half of the midbrain of csf1ra+/+, csf1raA784V/+, csf1raex17∆5/+ and csf1raex17∆5/A784V at 3 dpf. White arrows show small NR+ dots in the region where astrocytes reside b Representative images of LysoTracker (magenta) staining of csf1ra+/+, csf1raA784V/+, csf1raex17∆5/+ and csf1raex17∆5/A784V in a her4:GFP (green) background, visualizing radial astrocytes, in vivo at 3 dpf. c Quantifications of the number of LT+ inclusions within radial astrocytes in the midbrain at 3 dpf in csf1ra+/+ (n=7), csf1raA784V/+ (n=10), csf1raex17∆5/+ (n=5) and csf1raex17∆5/A784V (n=11) d Schematic representation of the zebrafish midbrain (mb), hindbrain (hb) and indicated ROI. One way ANOVA was preformed to test for significance (p<0.05). * p<0.05 ** p<0.01 *** p<0.001. Error bars represent SD. Scale bar equals 20 μm.Supplementary Fig. 8 Lysosomal inclusion are exclusively present in radial astrocytes and the midbrain a Representative images of slc1a2b (yellow) transgenic larvae (WT and csf1raV614M/V614M) treated with LysoTracker (magenta) showing the absence of LT+ inclusions in the hindbrain and the spinal cord, and in the midbrain at 2 dpf b Representative images of elavl3:GFP+ neurons (green) transgenic larvae (csf1raV614M/V614M) treated with LysoTracker (magenta) showing the absence of LT+ inclusions in neurons at 3 dpf. Scale bars equal 10 μm. (PDF 296754 KB)

Supplementary file2 Clinical and demographic characteristics of ALSP and control donors (XLSX 11 KB)

Supplementary file3 Overview of all pathogenic ALSP variants identified in patients(XLSX 31 KB)

Supplementary file4 Differentially expressed genes in the occipital gyrus of ALSP patients (XLSX 9483 KB)

Supplementary file5 GO pathway analysis based on RNA seq results (XLSX 14 KB)

Supplementary file6 Differentially expressed proteins in the occipital gyrus of ALSP patients(XLSX 3282 KB)

Supplementary file7 Comparison of differentially expressed genes and proteins in the occipital gyrus of ALSP patients to published datasets (XLSX 43 KB)

Supplementary file8 In vivo timelapse video of pHrodo-labelled myelin (magenta) injected csf1ra+/+, csf1raV614M/+, and csf1raV614M/V614M larvae (n = 3/group) in a transgenic radial astrocyte (her4:GFP, green) background, 18 hpi until 21 hpi, interval: 3 min (AVI 225,797 KB)
