# Supplementary material for: Dominant-acting CSF1R variants cause microglial depletion and altered astrocytic phenotype in zebrafish and adult-onset leukodystrophy
Source: Acta Neuropathol. 2022 Jun 17;144(2):211–39. doi: 10.1007/s00401-022-02440-5 (PMC9288387; doi:10.1007/s00401-022-02440-5)
Supplement: Supplementary file 1 — Supplementary file1 Neuropathological examination of POLD patients and extra characterizations of microglia a Representative images of LFB and PAS staining of the frontal gyrus of POLD diagnosed patients (n=4), showing myelin loss and pigmented glia in the white matter b, c Representative NF (b) and HE (c) IHC images of the frontal gyrus of POLD diagnosed patients (n=4), showing axonal spheroids and pigmented glia in the white matter d Representative CD163 IHC images of the frontal gyrus of controls (n=3), intermediate-stage ALSP patients (n=2) and late-stage ALSP patients (n=6) in grey (top row) and white matter (bottom row) e Representative CD163 IHC images of the frontal gyrus of intermediate-stage ALSP patients showing clustered distribution of CD163+ cells in the white matter. Scale bars equal 50 μm (a, b, d), 100 μm (c) and 500 μm (e).Supplementary Fig. 2 Heterozygous ALSP-causing CSF1R missense variants cause defective proliferation of microglia a Representative image of mpeg1:GFP+ yolk sac macrophages (YSM) at 24 hpf. Dashed lines show outlines of the yolk sac (YS), the embryonic brain (EB) and the eye (E) b Quantification of mpeg1:GFP+ YSM on the yolk (left) and in the embryonic brain (right) at 24 hpf in csf1ra+/+ (n = 12) and csf1raV614M/+ (n=14) larvae c Maximum projection of a timelapse (32-47 hpf, 15 min interval) showing mpeg1:GFP+ YSM migration from the yolk sac into the embryonic brain d Quantification of mpeg1:GFP+ cells on the yolk sac (left) and in the embryonic brain (right) over time (32-47 hpf) in csf1ra+/+, csf1raA784V/+ and csf1raV614M/+ (n=4/group) e Representative images of single mpeg1:GFP+ microglia proliferating in vivo in the midbrain of zebrafish larvae, in a timelapse between 54-72 hpf f Quantification of proliferating microglia in the midbrain of csf1ra+/+ and csf1raV614M/+ larvae between 54 and 72 hpf (n=3/group). Eye (E), embryonic brain (EB), yolk sac (YS). One way or two way ANOVA, and Student t-test was preformed to test for si [file 401_2022_2440_MOESM1_ESM.pdf]

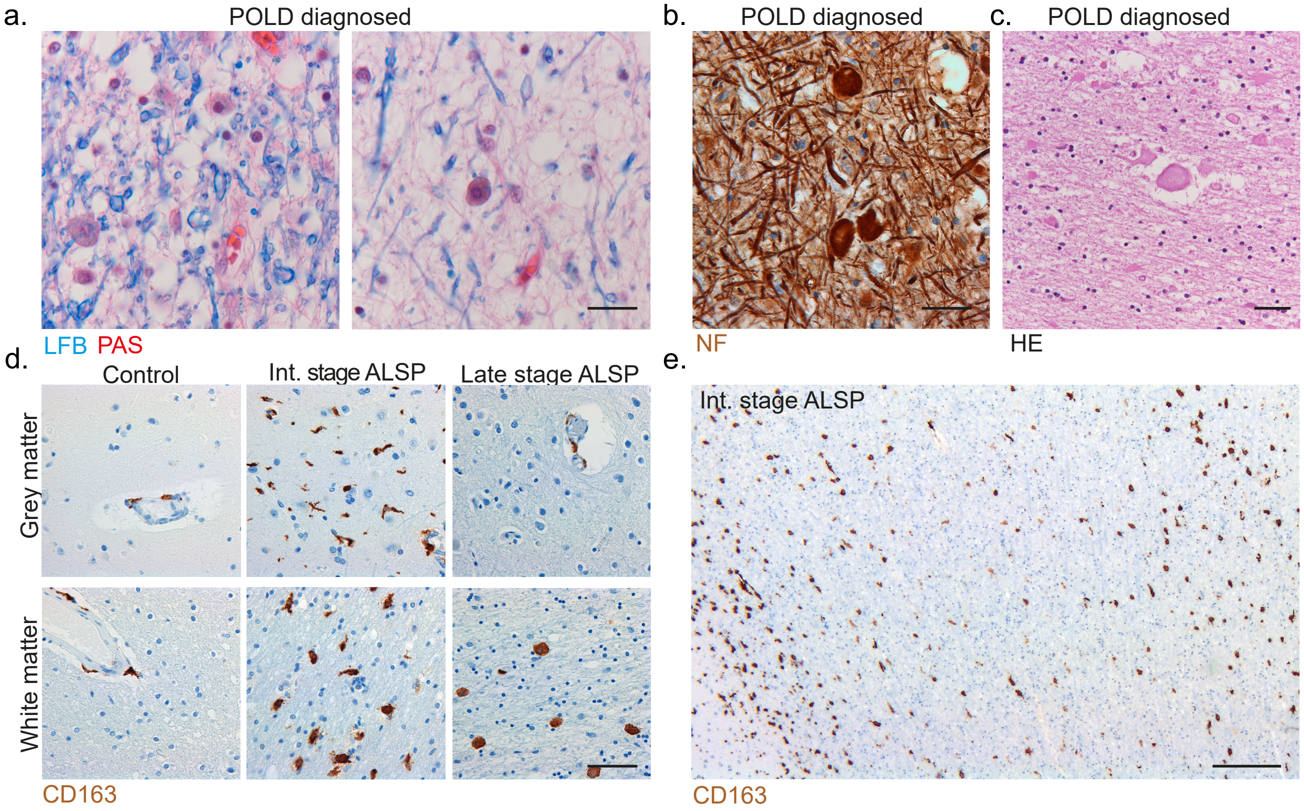
a.
POLD diagnosed
LFB PAS
b.
POLD diagnosed
NF
c.
POLD diagnosed
HE
d.
Control
Int. stage ALSP
Late stage ALSP
Grey matter
White matter
CD163
e.
Int. stage ALSP
CD163

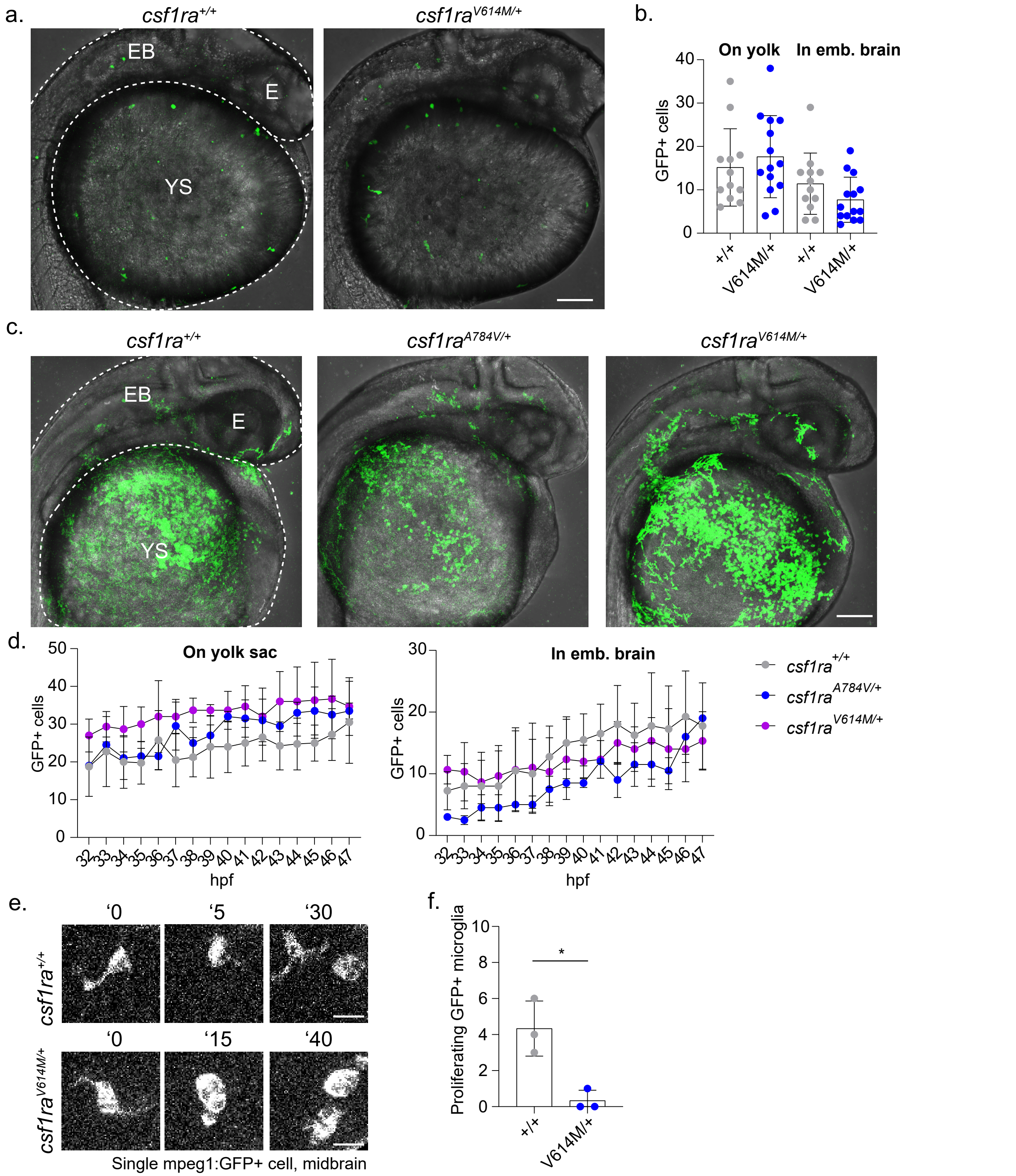

a.
csf1ra+/+
csf1raV614M/+
EB
E
YS
b.
On yolk
In emb. brain
GFP+ cells
40
30
20
10
0
+/+
V614M/+
+/+
V614M/+
c.
csf1ra+/+
csf1raA784V/+
csf1raV614M/+
EB
E
YS
d.
On yolk sac
In emb. brain
GFP+ cells
hpf
32 33 34 35 36 37 38 39 40 41 42 43 44 45 46 47
csf1ra+/+
csf1raA784V/+
csf1raV614M/+
e.
'0
'5
'30
'0
'15
'40
csf1ra+/+
csf1raV614M/+
Single mpeg1:GFP+ cell, midbrain
f.
Proliferating GFP+ microglia
10
8
6
4
2
0
*
+/+
V614M/+

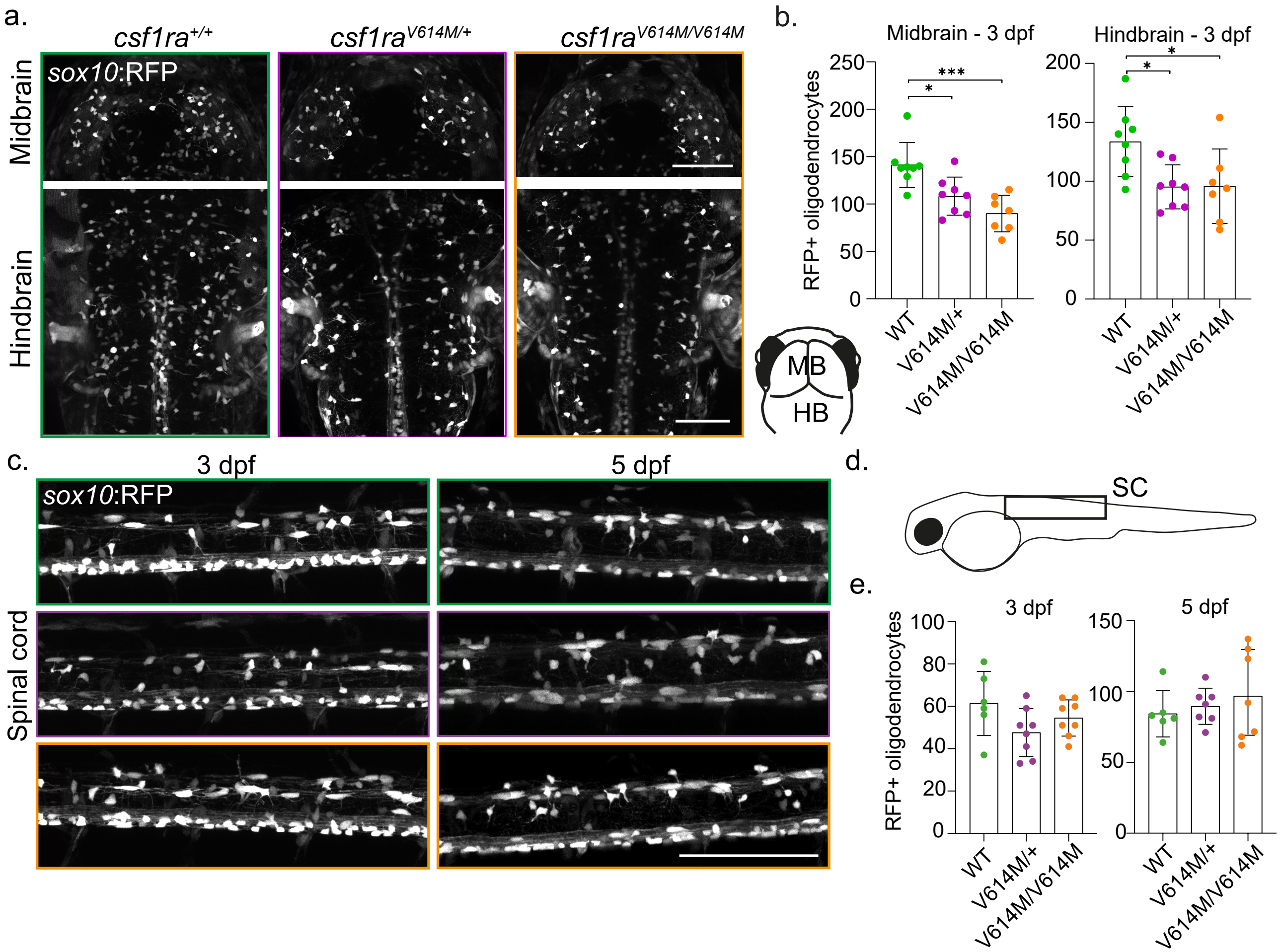

a.
csf1ra+/+
csf1raV614M/+
csf1raV614M/V614M
sox10:RFP
Midbrain
Hindbrain
b.
Midbrain - 3 dpf
Hindbrain - 3 dpf
RFP+ oligodendrocytes
250
200
150
100
50
0
***
*
WT
V614M/+
V614M/V614M
MB
HB
c.
3 dpf
5 dpf
Spinal cord
d.
SC
e.
3 dpf
5 dpf
RFP+ oligodendrocytes

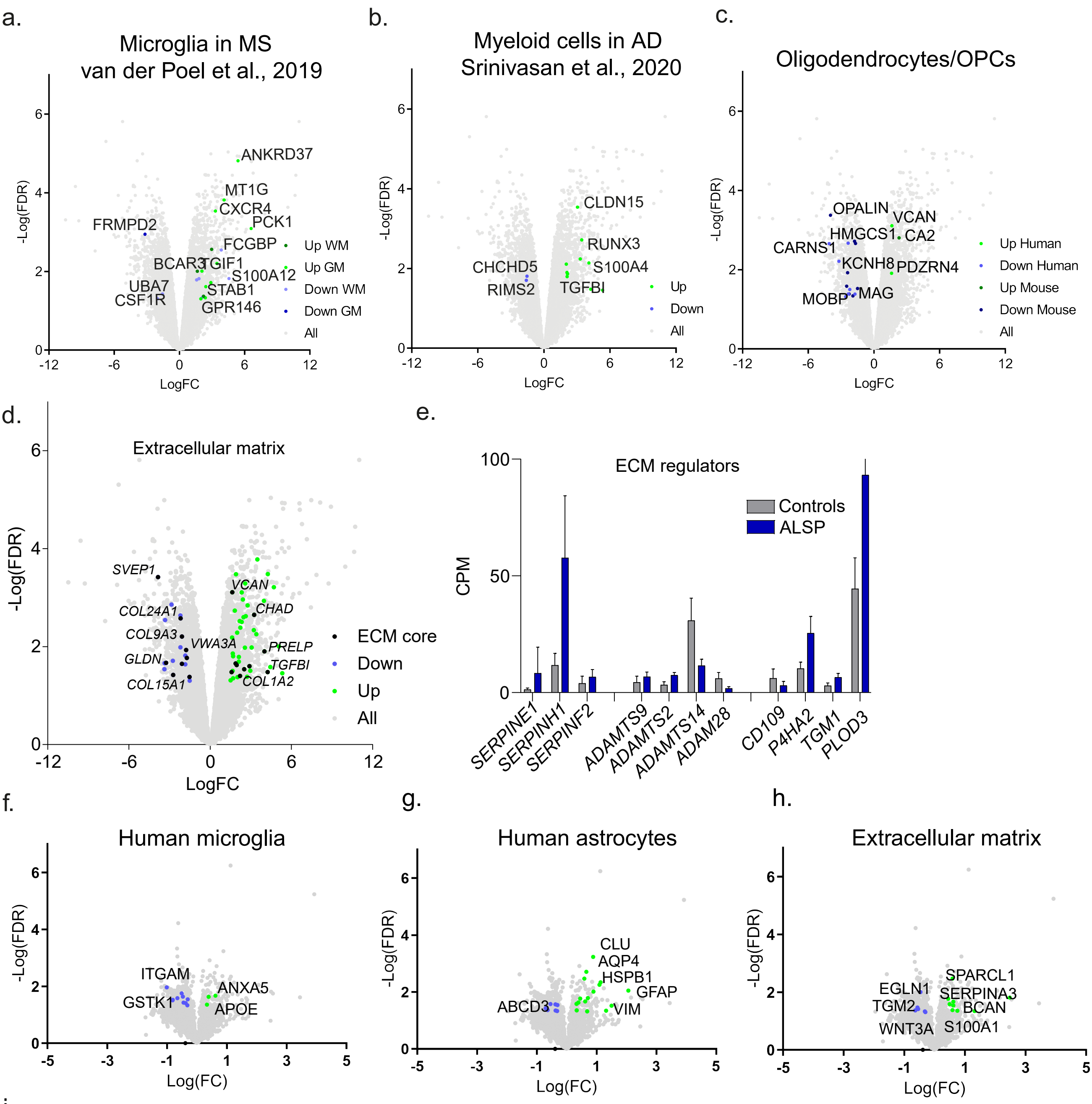

i.

Fresh frozen	Sex	A.o.Di. (y)	A.o.De. (y)	PM delay (h)	CSF1R variants
ALSP 1	M	46	49	08:30	c.2381T>C, p.(Ile794Thr)
ALSP 2	M	45	52	05:10	c.1889T>G, p.(Leu630Arg)
CONTROL 6	M	-	56	09:15	-
CONTROL 7	M	-	55	07:15	-

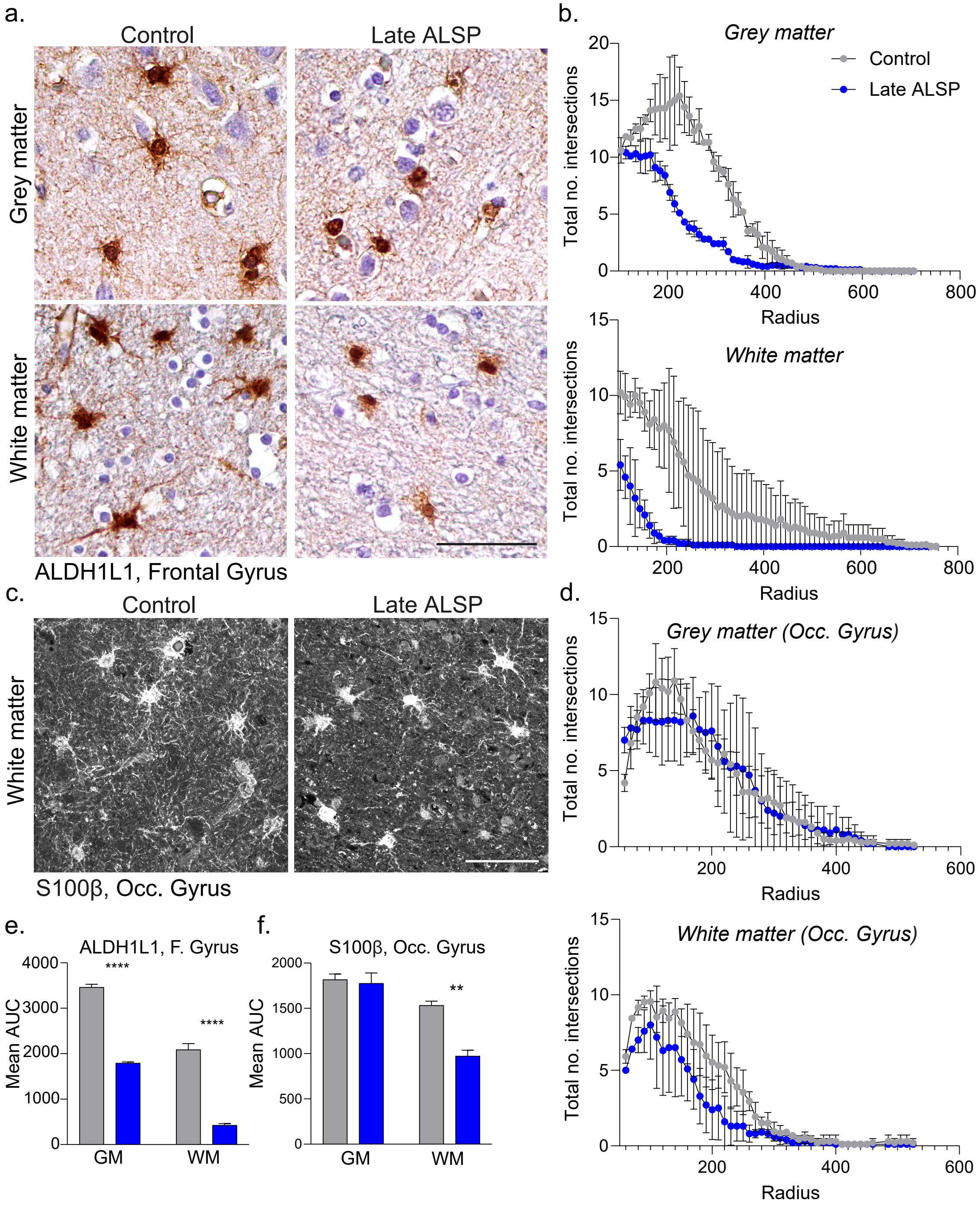
a.
Control
Late ALSP
Grey matter
White matter
ALDH1L1, Frontal Gyrus
b.
Grey matter
Control
Late ALSP
Total no. intersections
Radius
White matter
c.
Control
Late ALSP
White matter
S100β, Occ. Gyrus
d.
Grey matter (Occ. Gyrus)
White matter (Occ. Gyrus)
e.
ALDH1L1, F. Gyrus
Mean AUC
****
GM
WM
f.
S100β, Occ. Gyrus
**

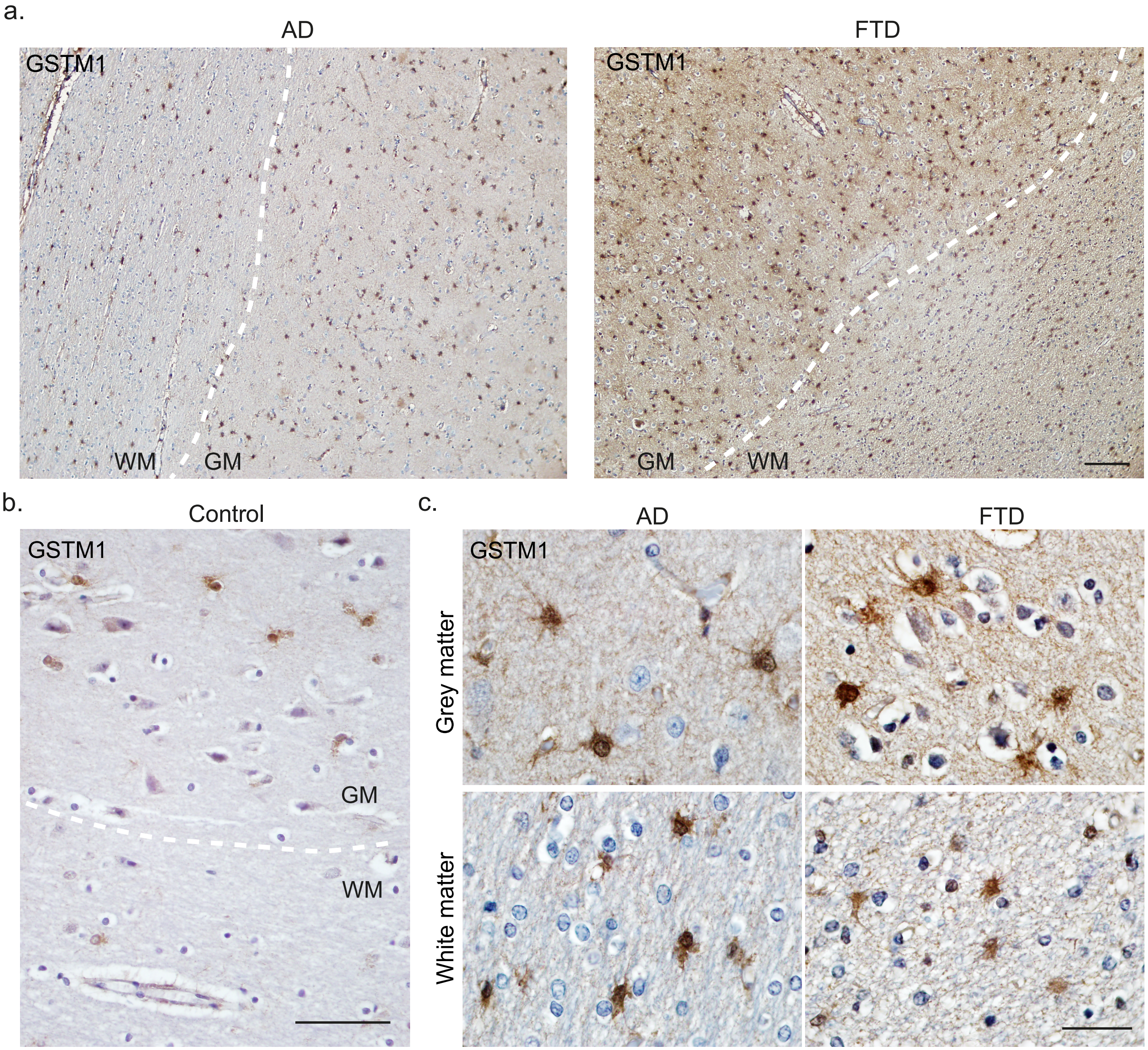

d.

Diagnosis	Sex	Age (y)	PM delay (h)
FTD (TAU)	F	59	5:50
FTD (TAU)	M	51	4:25
AD	M	71	5:55
AD	F	75	15:00
Control	M	49	6:15

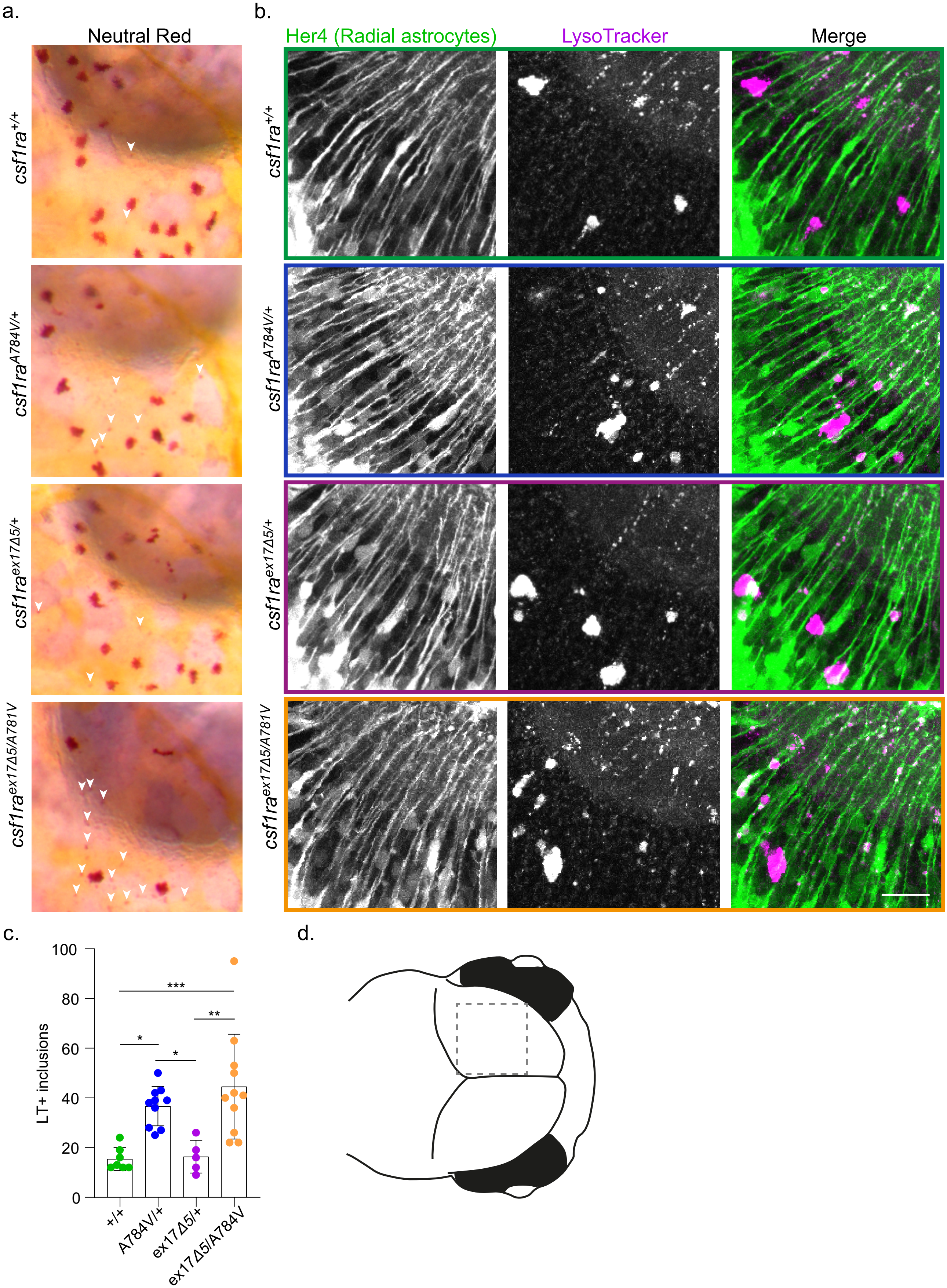

a.
Neutral Red
csf1ra+/+
csf1raA784V/+
csf1raex17Δ5/+
csf1raex17Δ5/A781V
b.
Her4 (Radial astrocytes)
LysoTracker
Merge
csf1ra+/+
csf1raA784V/+
csf1raex17Δ5/+
csf1raex17Δ5/A781V
c.
100
80
60
40
20
0
LT+ inclusions
***
**
*
*
+/+
A784V/+
ex17Δ5/+
ex17Δ5/A784V
d.

a.

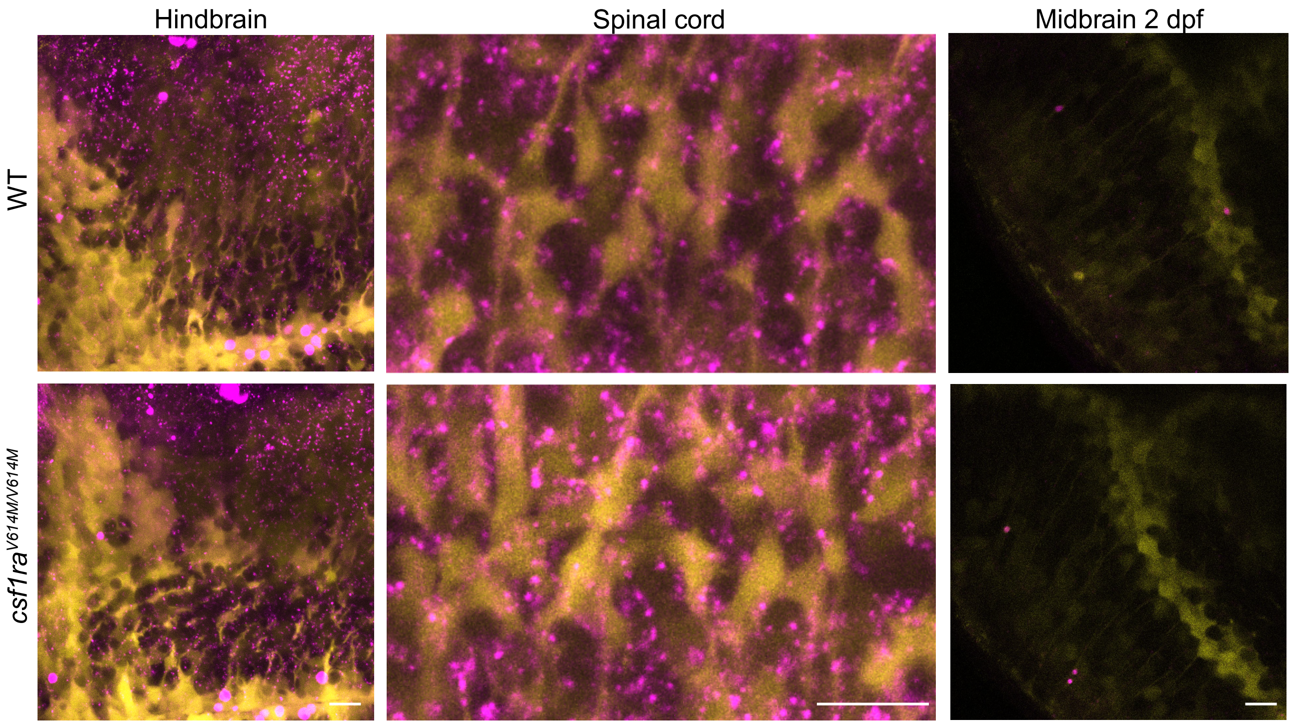

Hindbrain
Spinal cord
Midbrain 2 dpf
WT
csf1ra V614M/V614M

b.

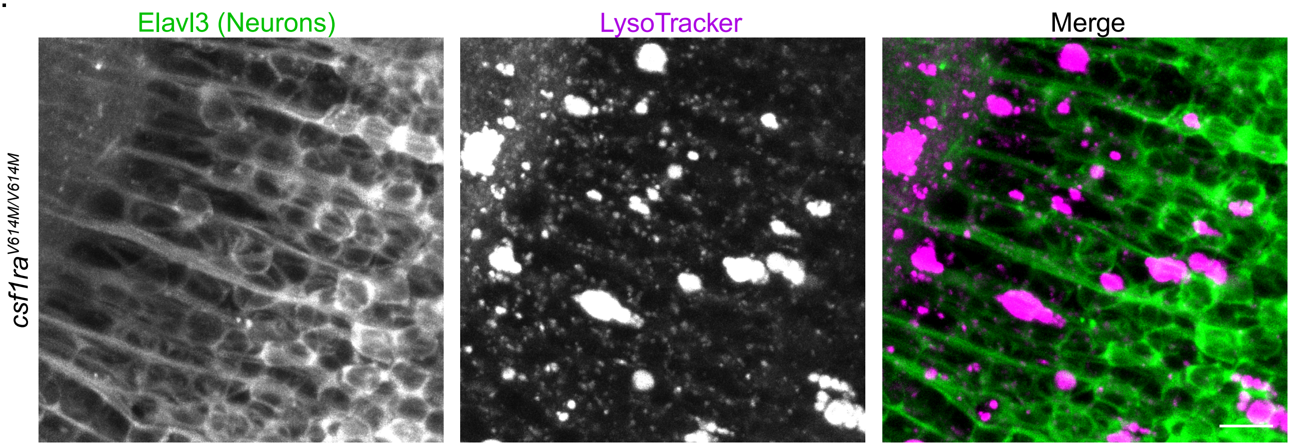

Elavl3 (Neurons)
LysoTracker
Merge
csf1ra V614M/V614M
